# Continuous Additive Manufacturing using Olefin Metathesis

**DOI:** 10.1002/advs.202200770

**Published:** 2022-03-10

**Authors:** Jeffrey C. Foster, Adam W. Cook, Nicolas T. Monk, Brad H. Jones, Leah N. Appelhans, Erica M. Redline, Samuel C. Leguizamon

**Affiliations:** ^1^ Sandia National Laboratories Albuquerque NM 87185 USA

**Keywords:** additive manufacturing, dual‐wavelength, olefin metathesis, photosensitizer, stereolithography

## Abstract

The development of chemistry is reported to implement selective dual‐wavelength olefin metathesis polymerization for continuous additive manufacturing (AM). A resin formulation based on dicyclopentadiene is produced using a latent olefin metathesis catalyst, various photosensitizers (PSs) and photobase generators (PBGs) to achieve efficient initiation at one wavelength (e.g., blue light) and fast catalyst decomposition and polymerization deactivation at a second (e.g., UV‐light). This process enables 2D stereolithographic (SLA) printing, either using photomasks or patterned, collimated light. Importantly, the same process is readily adapted for 3D continuous AM, with printing rates of 36 mm h^–1^ for patterned light and up to 180 mm h^–1^ using un‐patterned, high intensity light.

## Graphical abstract

Selective dual‐wavelength olefin metathesis polymerization enables the single exposure, topological printing of images and the 3D continuous additive manufacturing of structures from resin formulations based on dicyclopentadiene. Printing rates of 36 mm h^–1^ for patterned light and up to 180 mm h^–1^ using un‐patterned, high intensity light are shown.

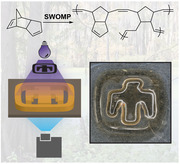

## 1 Introduction

The remarkable flexibility of 3D printing technologies enables rapid production of complex objects with designed internal features. Collectively referred to as additive manufacturing (AM), this suite of techniques is ideally suited for prototyping and customized manufacturing,^[^ ^1^ ^]^ and has been leveraged for the fabrication of products ranging from medical devices,^[^ ^2–^, ^4^ ^]^ to made‐to‐order athletic wear,^[^ ^5^ ^]^ to aerospace components.^[^ ^6^ ^]^ In particular, vat polymerization AM techniques such as stereolithography (SLA) have found broad industrial use.^[^ ^7^ ^]^ During SLA, a 3D object is produced layer‐wise through a series of cross‐sectional curing steps using a photopolymerizable resin. The shape of the resulting object is determined by the pattern of the incident light, and thus the potential geometry space for objects produced by SLA is vast. However, SLA and related methods rely, almost exclusively, on free radical polymerization (FRP) chemistry, limiting the diversity of available monomers (e.g., acrylates) and thus material properties.^[^ ^8–^, ^13^ ^]^

An additional limitation of SLA printing is the time‐consuming delamination and recoating steps between each printed layer, restricting printing speed to millimeters or centimeters per hour.^[^ ^14^ ^]^ Continuous liquid interface production (CLIP) has addressed this limitation by creating a layer of inhibited polymerization within the photoresin adjacent to the projection window such that delamination and recoating is unnecessary.^[^ ^15^ ^]^ More recently, dual‐wavelength printing systems have been developed for FRP that employ photo‐orthogonal initiation and inhibition chemistries to maximize printing speed (e.g., 2000 mm h^–1^).^[^ ^14^, ^16^ ^]^

To expand the design space of continuous AM, we explored the use of ring‐opening metathesis polymerization (ROMP) coupled with dual‐wavelength SLA. Polymers produced by ROMP have a higher thermomechanical and chemical property ceiling compared with polyacrylates,^[^ ^17^, ^18^ ^]^ and can be tailored to include sidechain and backbone heterogeneity in terms of both configuration and composition.^[^ ^19–^, ^21^ ^]^ For example, analogues of polyethylene,^[^ ^22^, ^23^ ^]^ polyurethane, polyamide,^[^ ^24^ ^]^ poly(acetylene),^[^ ^25^ ^]^ and poly(*p*‐phenylene vinylidene)^[^ ^26^ ^]^ have all been prepared via by ROMP of cyclic olefin monomers. While photopolymerization strategies have been developed for ROMP,^[^ ^27–^, ^31^ ^]^ ROMP‐based AM using decomposition/deactivation chemistry has yet to be reported. As such, we were motivated to develop a dual‐wavelength vat photopolymerization process to combine the chemical and structural diversity of ROMP with the speed of continuous AM.

**Figure 1:**
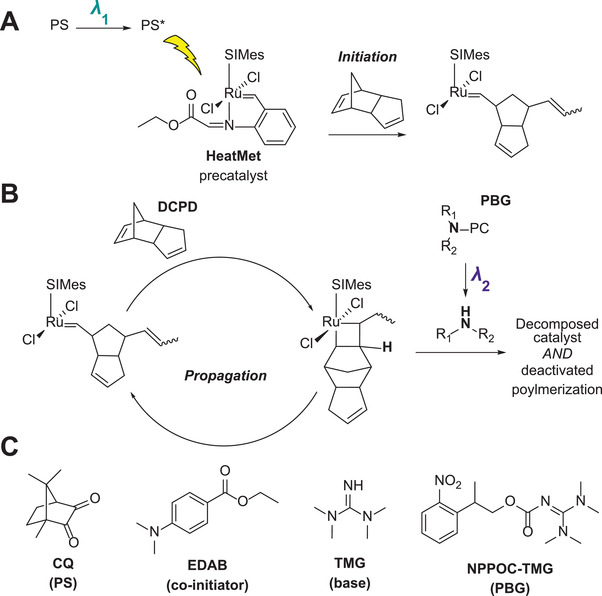
General overview of SWOMP chemistry using HM as the catalyst and DCPD as the monomer. a) Irradiation at *λ*_1_ initiates the catalyst via photosensitization, while b) generation of amine base by PBG photolysis at *λ*_2_ decomposes the catalyst and deactivates polymerization. c) Formulation components used in optimized photoresin. PC, photo‐cage; SIMes, 1,3‐Bis(2,4,6‐trimethylphenyl)‐4,5‐dihydroimidazol‐2‐ylidene; CQ, camphorquinone; EDAB, ethyl‐4‐(dimethylamino) benzoate; TMG, 1,1,3,3‐tetramethyl guanidine; NPPOC, 3‐nitrophenylpropyloxycarbonyl; DCPD, dicyclopendatiene.

Our recent work identified HeatMet (HM) (**Figure** **1**) as a photo‐active olefin metathesis catalyst to photopolymerize dicyclopentadiene (DCPD) under UV irradiation to yield a high‐performance thermoset material.^[^ ^32^, ^33^ ^]^ To adapt our HM/DCPD system to a dual‐wavelength photo‐activation/photo‐deactivation approach, we explored the use of HM in combination with a photobase generator (PBG). Ru‐based metathesis catalysts are susceptible to degradation via metallacyclobutane deprotonation using phosphines or amines^[^ ^34^ ^]^; thus, we envisioned that PBG photolysis could be leveraged to mediate polymerization deactivation. The use of a dual‐wavelength approach would allow for volumetric patterning while simultaneously fostering rapid printing speeds. In this contribution, we report the development of selective dual‐wavelength olefin metathesis polymerization (SWOMP) and use it to implement continuous SLA. Based on the versatility of ROMP and the broad scope of chemistries amenable to polymerization, as well as the high impact strength and excellent chemical and thermal resistance of DCPD thermosets, we anticipate these findings will underpin the creation of bespoke printed components with applications ranging from automotive^[^ ^18^ ^]^ or aerospace^[^ ^35^ ^]^ components to membranes^[^ ^17^ ^]^ to degradable materials.^[^ ^36^ ^]^

## 2 Results and Discussion

The implementation of SWOMP necessitated the development of photo‐orthogonal initiation and deactivation chemistries relevant to metathesis (Figure 1). In this scenario, irradiation of the catalyst at one wavelength, *λ*_1_, would promote initiation while irradiation at a second wavelength, *λ*_2_, would decompose the catalyst and thus deactivate polymerization. Note that we refer to polymerization deactivation as a dramatic cessation of monomer conversion. For SWOMP, deactivation is mediated by catalyst decomposition and reduction of the overall concentration of propagating catalyst species and re‐activation by initiation of further HM. Previous work by Lemcoff and co‐workers has demonstrated two‐wavelength olefin metathesis; however, deep UV light and long irradiation times were required for catalyst decomposition—conditions that are unsuitable for AM applications.^[^ ^30^, ^37^ ^]^ To create a deactivation layer, the kinetics of deactivation—a two‐step process involving photolysis of a PBG and subsequent reaction of the liberated base with the active Ru catalytic species—would have to compete with the rates of catalyst initiation and propagation to effectively inhibit curing. We also needed to consider the overall polymerization rate profile, as this factor was directly related to the maximum printing speed that could be achieved. Thus, several parameters were identified that required optimization: initiator absorption wavelength, initiation rate, PBG absorption wavelength, decomposition/deactivation chemistry, and overall polymerization kinetics. The latter is primarily determined by catalyst identity and monomer concentration, and these were fixed across all experiments. The other parameters were systematically evaluated by conducting polymerizations using resins formulated with different photosensitizers (PSs), PBGs, and stoichiometries. DCPD was chosen as primary resin component based on its high ring strain and the excellent thermomechanical properties of its resulting material; 5‐ethylene‐2‐norbornene (ENB) was used as a comonomer to depress the melting point of the mixture to produce a low‐viscosity printing resin^[^ ^29^ ^]^ and allow rapid liquid infill, as resin viscosity is a key factor in achieving maximum printing rates.^[^ ^14^, ^38^ ^]^

To modulate initiator absorption profile and initiation rate, camphorquinone (CQ), 2‐isopropylthioxanthone (ITX), and benzil, were evaluated as PSs for HM and ethyl‐4‐(dimethylamino) benzoate (EDAB) was used as a co‐initiator. To the best of our knowledge, this represents the first report of using CQ or benzil to photosensitize Ru‐based metathesis. Polymerizations were carried out in the presence of HM alone, HM+PS, or HM+PS+EDAB, and were monitored by FT‐IR spectroscopy to determine monomer conversion and UV‐rheology to measure cure behavior. As shown in Figure S7 (Supporting Information), low conversion was obtained for HM in the absence of PS under the experimental conditions; however, addition of PS+EDAB resulted in increased conversion, polymerization rate, and gelation within the experimental timeframe. Additionally, the presence of PSs facilitated the use of longer irradiation wavelengths to initiate the polymerizations. HM alone initiated most efficiently at 365 nm, whereas the polymerization could be initiated at 405 nm in the presence of ITX or benzil, or at 475 nm when using CQ (Figure S8, Supporting Information).

Next, a series of amines (aniline, *n*‐butylamine, cyclohexylamine, piperidine, and tetramethyl guanidine, TMG) were evaluated for their capability to decompose the active HM‐derived catalyst species. These amines were rationally selected to elucidate the influences of nucleophilicity and basicity on catalyst decomposition and were amenable to photo‐caging. Two possible pathways of activity loss via catalyst decomposition by bases have been reported: 1) direct nucleophilic attack at the Ru carbene by phosphine or nitrogen and 2) metallacyclobutane deprotonation.^[^ ^39^ ^]^ Regardless of mechanism, treatment of Ru catalyst with excess amine was anticipated to trigger decomposition and polymerization deactivation. To evaluate this theory, polymerizations were carried out with the HM+Benzil+EDAB system in the presence of 1 equiv of amine under 405 nm light irradiation and monomer conversion was again monitored by FT‐IR spectroscopy (Figure S9A, Supporting Information). Amine nucleophilicity did not appear to influence monomer conversion or conversion rate, as evident in comparisons of amines of similar basicity (i.e., *n*‐butylamine, cyclohexylamine, and piperidine). In contrast, monomer conversion was observed to decrease linearly with increasing amine p*K*_a_, with TMG acting as the most efficient decomposer/deactivator (Figure S9B, Supporting Information).

Further insight into the deactivating effect of amines was gained using UV–vis spectroscopy. HM was mixed with 10 equiv TMG in dichloroethane solution in the presence or absence of monomer. Norbornene (NBE) was utilized as the monomer in this case to prevent gelation within the cuvette. As shown in Figure S10 (Supporting Information), no catalyst decomposition was observed in the presence of TMG either in the dark or with 365 nm irradiation, and polymerization readily occurred in the absence of TMG under 405 nm irradiation. In contrast, a decrease in the absorbance at *λ* ≈320 nm associated with the metal ligand charge transfer (MLCT) band signified carbene loss and thus catalyst decomposition when HM, NB, and TMG were all mixed and the light turned on.^[^ ^40^ ^]^ Moreover, no polymerization was evident under these conditions. These data suggest that amine basicity determined decomposition and deactivation efficiency in our system and that catalyst initiation was required before decomposition could occur. Both factors pointed toward metallacyclobutane deprotonation as the primary mechanism of decomposition (Scheme S1, Supporting Information). TMG was utilized as the decomposing species in subsequent experiments based on its superior efficiency.

PBGs supply a steady concentration of base—typically an amine—via photolysis of a protecting group. Of the numerous photo‐protecting groups reported, nitrobenzyl derivatives are perhaps the most versatile and synthetically accessible.^[^ ^41–^, ^44^ ^]^ These compounds typically undergo photolysis upon irradiation with UV light (Scheme S2, Supporting Information), and their release half‐lives can be tuned via chemical modification. We synthesized a series of three PBGs based on the 2‐nitrobenzyl moiety and using TMG as the base: 2‐nitrobenzyl TMG carbamate (NB‐TMG), 4,5‐dimethoxy‐2‐nitrobenzyl TMG carbamate (NVOC‐TMG), and 2‐(2‐nitrophenyl)propyl TMG carbamate (NPPOC‐TMG). Experimental details and characterization data are provided in the Supporting Information, including UV–vis spectra, which highlight minimal absorbance at ≥405 nm necessary for dual‐wavelength selectivity with the chosen PSs (Figure S11, Supporting Information).

**Figure 2:**
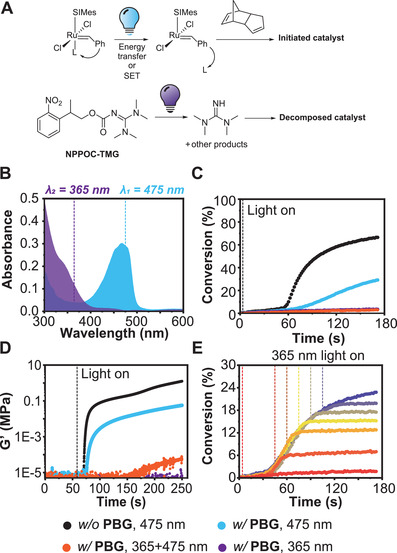
Optimized photoresin for SWOMP. a) Generalized schematic of photoinitiation and photo‐decomposition chemistries promoted by blue or UV light, respectively. b) UV–vis spectra demonstrating the photo‐orthogonality of the photosensitization (CQ, light blue spectrum) and photo‐decomposition (NPPOC‐TMG, purple spectrum) chemistries employed. Spectra were collected for the individual compounds at 0.01 mg mL^–1^ in CH_2_Cl_2_ solution. c) Polymerization kinetics as measured by FT‐IR spectroscopy at 1573 cm^–1^ for optimized photoresin irradiated with 475 nm light in the absence of PBG (black circles) and with PBG at 475 nm (blue circles), 365 nm (purple circles), and both wavelengths (orange circles). d) Evolution of modulus over time for the same resin formulation and irradiation wavelengths as in (c). e) Polymerization deactivation by turning on the 365 nm light at different times (*t* = 0, 45, 60, 75, 90, or 105 s) after initiation as compared to polymerization in the absence of 365 nm light (blue). The dashed lines represent the time at which the 365 nm light was turned on with the various colors corresponding to the separate kinetic traces as measured by FT‐IR. The 475 nm light was turned on at *t* = 0 s and was left on throughout the duration of the experiments. [DCPD]/[NPPOC‐TMG]/[HM] = 5000:10:1 was used for these experiments with 0.5 wt.% CQ and 1 wt.% EDAB.

To evaluate the orthogonality of the various PSs and PBGs, FT‐IR spectroscopy and UV‐rheology were used to monitor polymerization progress of DCPD by HM in combination with a PS and a PBG. Experiments were conducted under 365 nm irradiation to ensure efficient catalyst decomposition and deactivation, 405/475 nm irradiation (depending on the PS) to evaluate the influence of the PBGs on catalyst initiation, or both 365 nm and 405/475 irradiation to simulate the environment of the deactivation layer under printing conditions. Ideally, the presence of PBG in the photoresin formulation would have little influence on the rate and ultimate conversion of the polymerization under 405/475 light irradiation, whereas 365 nm light irradiation (or a combination of both initiation and decomposition wavelengths) would act to deactivate polymerization. All PBGs were found to effectively inhibit polymerization when 365 nm or a combination of 365 + 405/475 nm light were used, regardless of the selected PS (Figures S12 and S13, Supporting Information). It should be noted that limited deactivation was observed in all cases when exclusively irradiated at 405/475 nm, likely attributable to partial sensitization of the PBG by the respective PS.^[^ ^43^, ^45^ ^]^ However, NPPOC‐TMG had the least significant impact on monomer conversion under initiating conditions, and resin formulated with this PBG possessed the shortest incubation times for the onset of gelation. Based on these findings, and the relatively higher absorption maximum of CQ relative to the other PSs, the CQ+EDAB+NPPOC‐TMG resin system was taken forward (**Figure** **2**).

Additional optimization experiments were carried out by varying formulation stoichiometry (Figures S14 and S15, Supporting Information). The relative quantities of CQ, EDAB, and NPPOC‐TMG were systematically varied, with [CQ]/[EDAB]/[NPPOC‐TMG]/[HM] = 10:20:15:1 giving the most optimal performance in terms of polymerization rate under 475 nm irradiation and deactivation efficiency with the 365 nm light on. We also investigated how rapidly the DCPD polymerizations became deactivated by 365 nm irradiation. Additional kinetic experiments were carried out using either 5, 10, or 15 equiv of NPPOC‐TMG and followed by FT‐IR spectroscopy. For this series, 475 nm light was turned on at the onset to initiate polymerization and then a 365 nm light source was turned on at various times in separate experiments to decompose the catalyst and thus deactivate polymerization. As shown in Figure 2E and Figure S16A (Supporting Information), monomer conversion in the presence of 5 equiv NPPOC‐TMG was arrested 20 s after the 365 nm light was turned on (conversion plateaued with near‐zero additional conversion), while the 10 equiv NPPOC‐TMG series responded within ≈10 s and the 15 equiv series almost instantaneously. The ultimate conversions achieved in each case could be correlated to the time at which the 365 nm light was turned on (Figure S16B, Supporting Information) and tended to decrease with increasing NPPOC‐TMG loadings. The fact that polymerization could be rapidly turned off when using 15 equiv of NPPOC‐TMG suggested that both high print resolution and print speed should be achievable using SWOMP chemistry.

**Figure 3:**
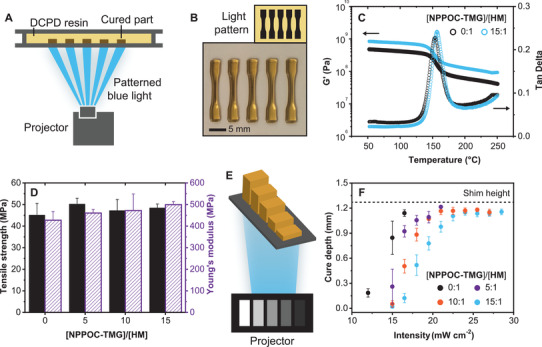
Summary of single wavelength SWOMP. a) Schematic of photopolymerization setup, wherein patterned blue light was projected into the photoresin from below. b) Optical photograph of PDCPD dogbones produced from the image shown above. c) DMA of PDCPD films prepared via SWOMP using the optimized resin with (blue circles) or without (back circles) 15 equiv of NPPOC‐TMG relative to HM. D) Measured tensile strengths (blue bars) and Young's moduli (purple shaded bars) of dogbones prepared by photopolymerization using the optimized resin and different amounts of NPPOC‐TMG. e) Schematic of projector image and resulting cured staircase structure used to determine cure depths. f) Measured cure depths obtained via photopolymerization by varying the projected light intensity and using optimized DCPD resins containing 0 (black circles), 5 (purple circles), 10 (orange circles), or 15 (blue circles) equiv of NPPOC‐TMG relative to HM. [DCPD]/[NPPOC‐TMG]/[HM] = 5000:15:1 was used for these experiments with 1 wt.% CQ and 2 wt.% EDAB.

We next considered whether the presence of PBG and its concentration might adversely affect the mechanical properties of the cured materials. PDCPD dogbones for use in mechanical testing were produced in a simulated printing environment using resin formulations with different loadings of NPPOC‐TMG. This setup, depicted in **Figure** **3**a, involved containment of the resin between two glass slides. The resin was then exposed to an image of blue light for 120 s, yielding cured parts with 3D dogbone geometries (Figure 3b). Polymer films were also produced using this method by projecting a large, rectangular image into the confined resin. The as printed objects were subjected to post‐cure at 250 °C for 30 min prior to analysis to fully consume unreacted DCPD monomer (Figure S17, Supporting Information). Thermal post‐cure is commonly employed for parts produced via DCPD polymerization and generally results in significantly higher glass transition, *T*_g_, values and improved mechanical performance.^[^ ^32^, ^46^ ^]^ As shown in Figure 3c (and Figures S18 and S19, Supporting Information), the presence of NPPOC‐TMG had no detrimental influence on glass transition temperature, *T*_g_, or temperature‐dependent storage modulus. Moreover, dogbones produced with variable NPPOC‐TMG loadings possessed nearly identical tensile strength and Young's modulus values compared with control samples that were cured without PBG. An additional advantage of DCPD polymerization via a ring‐opening mechanism was reduced shrinkage during cure. An average volumetric shrinkage value of 7 ± 2% was measured for photo‐cured PDCDP parts, consistent with volumetric shrinkage values for PDCPD reported in the literature and lower than the cure shrinkage of competing thermoset materials (typically 5–20%).^[^ ^17^, ^47^, ^48^ ^]^

Cure depth defines the depth to which light penetrates and cures the resin. Control over this parameter, in combination with deactivation height, underpins optimization of printing rates and must be known to minimize cure‐through when printing complex geometries.^[^ ^14^, ^49^ ^]^ Measurements to determine cure depth in our system were performed by projecting a gradient intensity image into the resin that produced a staircase‐like structure (Figure 3e), for which cured heights could be measured relative to the glass slide used as a projection window. We found that manipulation of cure depth could be readily achieved by varying both the incident light intensity and the concentration of NPPOC‐TMG in the resin formulations (Figure 3f), with higher blue light intensities increasing the depth of cure and higher NPPOC‐TMG loadings affecting the opposite result.

The data shown in Figure 3 exemplify the scope of photocuring using a single color; however, the addition of a second wavelength enabled catalyst decomposition chemistry and thereby expanded the capabilities of the photocuring system. As a simple demonstration, 2D geometries were readily produced using dual‐wavelength mask‐based stereolithography. For these experiments, the resin was illuminated with an un‐patterned background of blue light from below, and a UV light source, positioned above the resin, was patterned using a photomask (Figure S20, Supporting Information). Areas of resin exposed exclusively to blue light cured, whereas those regions additionally exposed to UV light did not cure and the residual resin was readily washed away. Complex shapes and features on the sub‐mm scale could be achieved using this method, as demonstrated in Figure S21 (Supporting Information).

**Figure 4:**
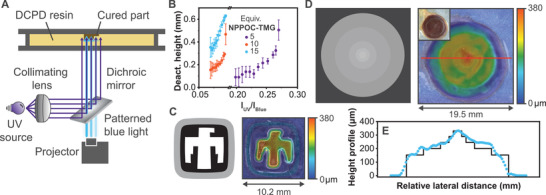
Summary of dual‐wavelength SWOMP. a) Schematic of setup for intensity‐patterned photopolymerization, wherein patterned, gray‐scaled blue light is superimposed with collimated UV light and projected into the resin to create a 3D object. b) Deactivation height as a function of UV/blue light intensity ratio for resins formulated with 5 (purple circles), 10 (orange circles), or 15 equiv (blue circles) of NPPOC‐TMG relative to HM. c,d) Multi‐level intensity images and corresponding topographical images of printed objects showing the heights of the different surface features. The inset in (d) is an optical image of the printed part. e) Measured (blue circles) and expected (black line) heights for the surface features obtained for the object in (d). The red line on the topographical image in (d) represents the profile path. Expected heights were calculated by subtracting the average deactivation height found in B from the spacer thickness (i.e., 635 µm) and lateral distances were scaled to match measured values. [DCPD]/[NPPOC‐TMG]/[HM] = 5000:15:1 was used for these experiments with 1 wt.% CQ and 2 wt.% EDAB.

When the resin is exposed to both UV and blue light from the same direction, a deactivation volume is created adjacent to the polymerization window in which polymerization does not occur. The thickness of this volume is defined by ratio of intensity of the two light sources and its geometry by the relative intensity at each point in space.^[^ ^14^ ^]^ Both parameters can be controlled across a defined area and up to the maximum cure depth by projecting a blue light image of variable intensity against an un‐patterned UV background. **Figure** **4**a shows a schematic of this process, wherein a variable intensity image (a grayscale image in this case) is superimposed upon a collimated UV light source using a dichromic mirror and then projected into the resin. To quantify the relationship between the relative intensities of the two light colors and the height of the deactivation volume, the optimized resin was exposed to a combination of a gradient image of blue light, similar to the pattern utilized in the cure depth experiments, and un‐patterned UV light such that the relative intensity of the two colors varied across the exposure area. As before, a staircase structure of cured material was obtained. This time, however, the object was cured to the far‐surface glass slide as opposed to the projection window. The height at each step corresponded to the inverse of the deactivation height, which is shown as a function of intensity ratio in Figure 4b. Here, the deactivation height appeared to scale exponentially with increased UV/blue light ratio, with higher UV light intensity needed for lower NPPOC‐TMG loadings. As such, printing rates could theoretically be controlled during continuous SWOMP by tuning the PBG concentration and/or the ratio of incident light intensities.^[^ ^14^ ^]^

**Figure 5:**
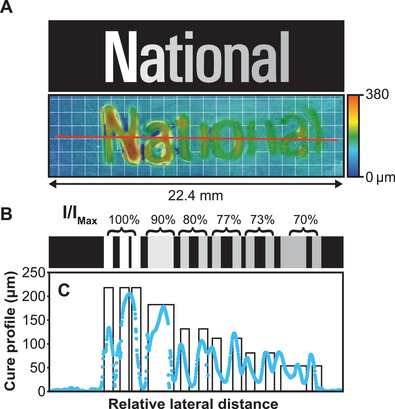
Additional SWOMP of “National” text, showcasing the multi‐dimensional precision of deactivation using multi‐intensity blue light patterning. a) Multi‐layer grayscale image and corresponding topographical image of tapered height “National” text. The grid represents 1 mm × 1 mm squares. b) Grayscale representation of the relative blue light intensities projected. c) Measured (blue circles) and expected (black line) heights for the surface features obtained for the object in (a). The red line on the topographical image in (a) represents the profile path. Expected heights were calculated by subtracting the deactivation height found using an exponential fit of the deactivation data from the spacer thickness (i.e., 500 µm) and lateral distances were scaled to match measured values. [DCPD]/[NPPOC‐TMG]/[HM] = 5000:15:1 was used for these experiments with 1 wt.% CQ and 2 wt.% EDAB.

A unique feature of dual‐wavelength SLA is the capability to produce complex 3D far‐surface features in a single exposure. As shown in Figure 4b, the volume of the deactivation layer can be directly controlled via the UV/blue light intensity ratio and was exploited to produce a staircase structure (vide supra). More complex structures could be readily achieved by simply changing the grayscale image used to project the blue light, which affects spatial control over the relative intensities of UV and blue light incident on each region of the resin. Multilayer Thunderbird and “wedding cake” objects were produced from single grayscale images with gradient shading (Figure 4c,d). Heights for the various layers, as determined by profilometry, closely matched expected values calculated using the deactivation height values (Figure 4e). This process was amenable to complex images, as demonstrated by the SLA printing of variable height text (**Figure** **5**). SLA printing of yet more complex objects were achieved simply by converting images to grayscale and projecting them into the resin (Figures S21 and S22, Supporting Information).

**Figure 6:**
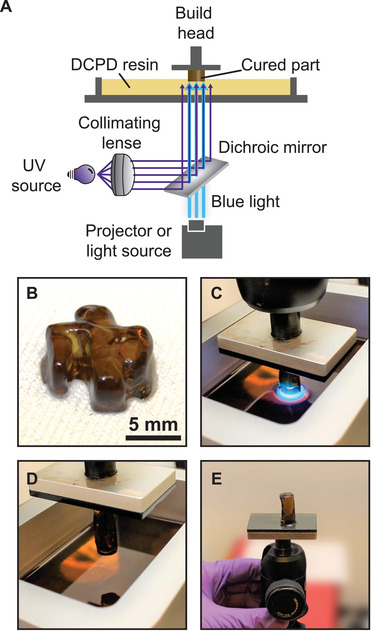
Summary of continuous SWOMP. a) Schematic of setup for continuous SLA, wherein patterned blue light is superimposed with collimated UV light and projected into the resin and an object forms on the build head, which becomes progressively taller as the build head is withdrawn. b) Optical image of a Thunderbird object obtained using a continuous SWOMP projector setup at a printing rate of 36 mm h^–1^. c–e) Photographs of cylindrical object obtained using a high‐intensity lamp setup and a printing rate of 180 mm h^–1^ during printing (c), immediately after printing (d), and inverted after removing from the printer (e). [DCPD]/[NPPOC‐TMG]/[HM] = 5000:15:1 was used for these experiments with 1 wt.% CQ and 2 wt.% EDAB.

As a proof of concept, continuous SLA was attempted using our dual‐wavelength ROMP system. Here, a build head was submerged into a vat of optimized photoresin using a similar illumination setup to the grayscale printing experiments (**Figure** **6**a). An initial resin height of ≈3 mm was used, which was >10× the thickness of the deactivation layer under the experimental conditions, as determined previously. To produce a 3D object patterned, blue light (30 mW cm^–2^) was superimposed against a UV flood (1.75 mW cm^–2^) and projected into the resin. The build head was then withdrawn at a rate of 36 mm h^–1^ to produce a 4 mm thick “Thunderbird” object (Figure 6b). This rate was determined to be optimal based on the measured intensity of the incident blue light. To further increase printing speed, the blue light projector and UV sources were replaced with an un‐patterned, multi‐wavelength, high intensity light source (475 nm@220 mW cm^–2^ and 365 nm@80 mW cm^–2^). Here, a 27 mm tall cylindrical object was produced at a rate of 180 mm h^–1^ during continuous SWOMP (Figure 6c–e, see Video S1, Supporting Information). Notably, this printing speed is substantially faster than conventional SLA.^[^ ^14^, ^15^ ^]^

## 3 Conclusion

The first demonstration of continuous AM using olefin metathesis employing a dual‐wavelength photo‐activation/photo‐decomposition and deactivation approach (SWOMP) as well as the first report of using CQ or benzil to photosensitize Ru‐based metathesis was shown. In addition to topologically complex objects produced using a selective wavelength photoresist approach, continuous SWOMP was developed to create complex, 3D objects using UV light in combination with patterned, multi‐intensity blue light. Importantly, the additional PS and PBG formulation components had no detrimental influence on the thermomechanical performance of the cured materials. Continuous printing rates were found to be competitive with existing continuous printing technologies based on FRP chemistry but substantially faster than traditional SLA. Further refinement of activation/deactivation chemistry is expected to enable the continuous printing of yet more complex objects at higher printing rates. The wavelength‐selective chemistry developed herein is expected to have broad implications for AM in terms of material and property selection and will inspire nascent dual‐wavelength processes.

## 4 Experimental Section

### Representative DCPD Resin Formulation

DCPD/ENB mixtures were first prepared at 5 wt.% ENB by adding DCPD melted at 40–50 °C to a glass jar containing ENB and agitating until fully mixed. Photoresin was then formulated using the DCPD/ENB mixture as follows: to a 125 mL Thinky cup was added 20 mg of HM (0.030 mmol, 1 equiv), 200 mg of CQ (1.2 mmol, 40 equiv), 400 mg of EDAB (2.1 mmol, 70 equiv), and 140 mg of NPPOC‐TMG (0.45 mmol, 15 equiv). CH_2_Cl_2_ was added in portions (≈1 mL total volume) to fully homogenize these components, consistent with established literature procedures.^[^ ^27^, ^30^, ^50^, ^51^ ^]^ Twenty grams of DCPD/ENB mixture was then added, and the resin was agitated to homogenize. The photoresin was used immediately after preparation.

### Selective Dual‐Wavelength Olefin Metathesis Polymerization

UV light from a high‐powered LED (*λ*_max_ = 365 nm, 1400 mA, Thorlabs #M365LP1) was collimated using an aspheric condenser lens [Ø2″; f = 32 mm; numerical aperture (NA), 0.76; Thorlabs, ACL50832U] and focused with an adjustable collimation adapter (Thorlabs, SM2F). A commercial DLP projector (Optoma ML750) was retrofitted to emit blue light by disconnecting the green and red LEDs. Light from the blue projection system was passed through a biconvex (Ø2″; f = 100 mm; NA, 0.76; Thorlabs, LB1630) lens to reduce the focal distance and superimposed with the UV light using a longpass dichroic mirror (Ø2″, 425‐nm cutoff; Thorlabs, DMLP425L). The intensity of the LEDs was controlled by adjusting the greyscale of an image in Microsoft PowerPoint. Light intensity at a given grey value was calibrated using a Thorlabs digital handheld optical power and energy meter console (PM100D). Spacers (two 0.635 mm stacked on each side) were clamped between two glass slides, and resin formulations were introduced between the slides via disposable pipette. The resin was irradiated for 3 min at various intensities using the projector. After, the slides were separated, rinsed with CH_2_Cl_2_, and allowed to dry in an oven at 50 °C to minimize post‐cure shrinkage. Final cure depth vales were measured using calipers. Similarly, deactivation depth was determined by using 0.635 mm spacers and subtracting the cure depth from 0.635 mm.

Topologically complex objects were created by projecting greyscale images into resin formulations sandwiched between glass slides using spacers of 0.503 mm or 0.635 mm for 3 min. After removal of one glass slide, the resulting polymer film was rinsed with CH_2_Cl_2_ prior to post‐curing in an oven initially for 2 h at 75 °C and then at 230 °C for an additional 30 min. Continuous SLA printing was performed using the setup described above, but glass slides were substituted with a vat possessing a transparent Teflon film (i.e., projection window). A build head (both projection window and build head were taken from a Kudo3D Micro SLA printer) attached to a programmable vertical stage (motorized linear stage, 100 mm travel, integrated controller, M4 and M6, catalog # FCL100) was used to continuously extrude the printed object from the vat. A Thorlabs CHROLIS 6‐wavelength high‐power LED source was used for continuous printing under high intensity irradiation.

## Conflict of Interest

The authors declare no conflict of interest.

## Author Contributions

The manuscript was written through contributions of all authors. All authors have given approval to the final version of the manuscript.

## Supporting information

Supporting Information

Supplemental Video 1

## Data Availability

The data that support the findings of this study are available in the supplementary material of this article.
