# Supplementary material for: Continuous Additive Manufacturing using Olefin Metathesis
Source: Adv Sci (Weinh). 2022 Mar 10;9(14):2200770. doi: 10.1002/advs.202200770 (PMC9108613; doi:10.1002/advs.202200770)
Supplement: Supplementary file 1 — Supporting Information [file ADVS-9-2200770-s001.pdf]

ADVANCED SCIENCE Open Access

Supporting Information

for *Adv. Sci.*, DOI 10.1002/advs.202200770

Continuous Additive Manufacturing using Olefin Metathesis

*Jeffrey C. Foster, Adam W. Cook, Nicolas T. Monk, Brad H. Jones, Leah N. Appelhans, Erica M. Redline and Samuel C. Leguizamon**

Supporting Information

“Continuous Additive Manufacturing Using Olefin Metathesis”

Jeffrey C. Foster, Adam W. Cook, Nicolas T. Monk, Brad H. Jones, Leah N. Appelhans, Erica M. Redline, and Samuel C. Leguizamon*

Sandia National Laboratories, Albuquerque, New Mexico 87185, United States *email: sleguiz@sandia.gov

Experimental Section

Materials and Methods

Materials

2-(2-Nitrophenyl)propyl chloroformate (NPPOC-Cl, ~95%), 4,5-dimethoxy-2-nitrobenzyl chloroformate (NVOC-Cl, 97%), 2-nitrobenzyl bromide (NB, 98%), 1,1′-carbonyldiimidazole (CDI, ≥ 90%), N,N,N′,N′-tetramethylguanidine (TMG, ≥ 99%), cyclohexylamine (≥ 99%), piperidine (99%), aniline (≥ 99.5%), and n-butylamine (99.5%), camphorquinone (CQ, ≥97%) and dicyclopentadiene (DCPD, ≥96%) were purchased from Sigma Aldrich. **HeatMet (HM)**, 5-ethylidene-2-norbornene (ENB), and ethyl 4-dimethylaminobenzoate (EDAB) were procured from Strem Chemicals, TCI America, and Oakwood chemicals, respectively. The photosensitizers 2-isopropylthioxanthone (ITX), and benzil were procured from aaBlocks. All chemicals were used as received unless otherwise specified.

Characterization Techniques

NMR spectroscopy. ^{1}H-NMR (500 MHz) and ^{13}C-NMR (125 MHz) spectra were recorded on a Bruker AVANCE III 500 spectrometer using chloroform-d ($CDCl_3$) as the solvent. Chemical shifts of protons are reported as δ in parts per million (ppm) and are relative to solvent residual peaks ($CDCl_3$: ^{1}H δ = 7.26 ppm; ^{13}C δ = 77.16).

Infrared spectroscopy. Mixtures of interest were introduced between NaCl salt plates (International Crystal Laboratories) separated by spacers (12.7 μm thick) to maintain constant sample thickness during polymerization. Each sample was placed in a Bruker-Tensor II FTIR spectrometer equipped with a horizontal transmission accessory (Pike) and spectra were collected

from 400 to 4000 cm^{-1} with 1 scan per acquisition for rapid data collection. Samples were irradiated at the wavelength(s) of interest (365 and 405 nm at 10 $mW \cdot cm^{-2}$ and 475 nm at 20 $mW \cdot cm^{-2}$), and irradiation was continuous for the duration of the data collection period. Irradiation was performed with a Thorlabs CHROLIS 6-Wavelength High-Power LED Source. Samples used for calculating conversion were formulated and measured with 3 replications. Conversions were calculated by fitting Gaussians (OriginPro 2020b. OriginLab Corp., Northampton, MA.) to peaks centered at 1573 cm^{-1} in the FTIR spectra and monitoring the disappearance of these peaks over time. Conversion at a given time, *C(t)*, is calculated by the following equation:

$$C(t) = \frac{\left(A_i - A(t)\right)}{A_i} * 100\%$$

Where A_i is the area of the Gaussian fitted to the initial absorbance peak and A_t is the peak area at a given time (t).

Differential scanning calorimetry. DSC was performed using a TA Instruments Q200 calorimeter. Approximately 5-10 mg of the sample was added to the bottom half of a standard DSC pan and the pan was then sealed. The sample was heated from room temperature to 250 °C, and this was followed by two additional cooling and heating cycles from 25-250 °C.

Thermogravimetric analysis. Thermal stability was evaluated using a TA Instruments Q5500 thermogravimetric analyzer. Samples were heated under nitrogen atmosphere (40 mL/min) at heating rate of 10 °C/min from room temperature to 600°C.

UV-Rheology. Rheology was performed on an ARES-G2 rheometer (TA Instruments) operating in small-amplitude oscillatory shear mode. Room temperature UV-rheology was performed using a Thorlabs CHROLIS 6-Wavelength High-Power LED Source and a UV accessory. Rheology was

performed with a parallel plate assembly, 2 Hz oscillation frequency, and a 1.0% strain amplitude. Exposure (60 mW·cm^{-2}) began after 60 seconds and was continuous for all runs. An approximate gap size of 0.4 mm was generally used. Additionally, the glass transition temperature was measured using a similar set up with a 3 °C/min ramp from room temperature to 250 °C and back.

UV-visible spectrophotometry. UV-visible spectrophotometry was performed using an Agilent Technologies Cary 60 UV–Vis spectrophotometer. Spectra were collected from 200 to 800 nm with 1 nm spacing on solutions using a 10 mm path length quartz cuvette. Irradiation of samples was provided by a collimated, LED-based illumination source (Thorlabs M365LP1-C1) with an emittance centered at 365 nm, used in combination with a current-adjustable LED driver (Thorlabs LEDD1B) to produce an intensity of 30 mW·cm^{-2}.

Tensile testing Tensile testing was performed using an electromechanical load frame (Instron 5982). Dogbone specimens (ASTM type V) were printed between two glass slides using 0.5 mm spacers and the projector to pattern dogbone images. The photo-cured dogbones were post-cured in an oven initially for two hours at 75 °C and then at 230 °C for an additional 30 minutes. Subsequently, the dogbones were subjected to uniaxial tension, and stress-strain curves were generated using low strain rates (3%·min^{-1}) to give convenient total test times.

Optical microscopy and surface profilometry. Images of printed coupons were acquired using a Kyence VHX-7000 digital microscope. Serial recording at 40x magnification and a 5-micron vertical pitch was employed to capture the full lateral and vertical profile of printed specimens. Topographical contour mappings were produced using Keyence's 3D imaging and analysis software package. The accuracy of the mappings were verified using a Bruker Dektak kXT contact stylus profilometer.

Synthetic Methods

Synthesis of 2-nitrobenzyl TMG carbamate (NB-TMG)

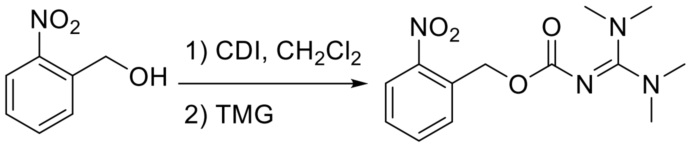

NB (1.0 g, 6.5 mmol, 1 equiv) was dissolved in 20 mL of CH_2Cl_2 in a 100 mL round bottom flask. The flask was placed in an ice bath. To the flask was added CDI (1.1 g, 6.5 mmol, 1 equiv) in one portion at 0 °C. Following this addition, the flask was removed from the ice bath and the reaction mixture was stirred for 1 h. TMG (0.82 mL, 6.5 mmol, 1 equiv) was then slowly added to the flask via syringe, and the reaction mixture was stirred at room temperature for 16 h. The reaction mixture was diluted with ~50 mL of CH_2Cl_2 and transferred to a separatory funnel. The organic mixture was washed with DI H_2O (2 x 50 mL) and brine, dried over Na_2SO4, and concentrated in vacuo. The residue was further purified on a silica gel column, eluting with a gradient from 0 – 10% MeOH in CH_2Cl_2. The purified product was isolated as a light-yellow oil that crystallized upon standing (0.96 g, 50% yield). **^{1}H NMR** (500 MHz, $CDCl_3$): δ (ppm) 8.07 (d, 1H), 7.77 (d, 1H), 7.60 (t, 1H), 7.41 (t, 1H), 5.51 (s, 2H), 2.88 (s, 6H), 2.87 (s, 6H). **^{13}C NMR** (500 MHz, $CDCl_3$): δ (ppm) 166.7, 159.6, 147.4, 134.7, 133.5, 128.9, 127.9, 124.7, 63.5, 39.9.

Synthesis of 4,5-dimethoxy-2-nitrobenzyl TMG carbamate (NVOC-TMG)

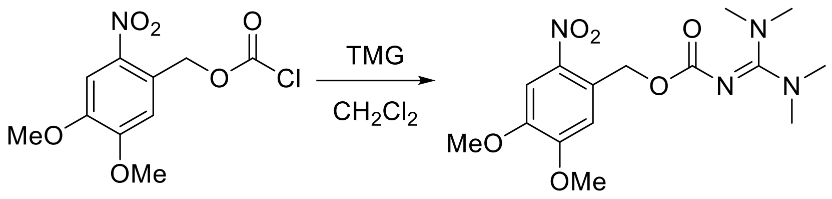

NVOC-Cl (1.1 g, 4.1 mmol, 1 equiv) was dissolved in 20 mL of CH_2Cl_2 in a 100 mL round bottom flask. The flask was placed in an ice bath. To the flask was added TMG (1.0 mL, 8.2 mmol, 2 equiv) dropwise via syringe at 0 °C. Following this addition, the flask was removed from the ice bath and the reaction mixture was stirred for 4 h. The reaction mixture was diluted with ~50 mL of CH_2Cl_2 and transferred to a separatory funnel. The organic mixture was washed with DI H_2O (2 x 50 mL) and brine, dried over Na_2SO4, and concentrated in vacuo. The residue was further purified on a silica gel column, eluting with a gradient from 0 – 10% MeOH in CH_2Cl_2. The purified product was isolated as a light-yellow oil that crystallized upon standing (0.90 g, 62% yield). **^{1}H NMR** (500 MHz, $CDCl_3$): δ (ppm) 7.70 (s, 1H), 7.20 (s, 1H), 5.52 (s, 2H), 3.94 (d, 6H), 2.88 (s, 12H). **^{13}C NMR** (500 MHz, $CDCl_3$): δ 166.8, 159.7, 153.6, 147.6, 139.6, 130.4, 109.8, 108.0, 63.7, 56.4, 39.9.

Synthesis of 2-(2-nitrophenyl)propyl TMG carbamate (NPPOC-TMG)

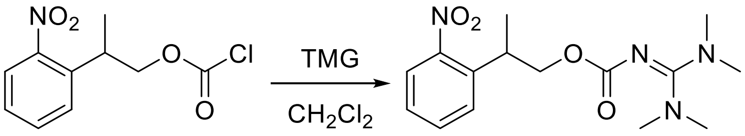

NPPOC-Cl (0.62 g, 2.5 mmol, 1 equiv) was dissolved in 20 mL of CH_2Cl_2 in a 100 mL round bottom flask. The flask was placed in an ice bath. To the flask was added TMG (0.64 mL, 5.1 mmol, 2 equiv) dropwise via syringe at 0 °C. Following this addition, the flask was removed from the ice bath and the reaction mixture was stirred for 16 h. The reaction mixture was diluted with ~50 mL of CH_2Cl_2 and transferred to a separatory funnel. The organic mixture was washed with brine (3x), dried over Na_2SO4, and concentrated in vacuo. The residue was further purified on a silica gel column, eluting with a gradient from 0 – 5% MeOH in CH_2Cl_2. The purified product was isolated as a light-yellow oil that crystallized upon standing (0.52 g, 64% yield). **^{1}H NMR** (500 MHz, $CDCl_3$): δ (ppm) 7.71 (d, 1H), 7.52 (m, 2H), 7.30 (td, 1H), 4.26 (d, 2H), 3.68 (m, 1H), 2.79 (s, 12H), 1.35 (d, 3H). **^{13}C NMR** (500 MHz, $CDCl_3$): δ (ppm) 166.2, 160.2, 150.4, 138.4, 132.5, 128.5, 127.0, 124.1, 68.4, 39.7, 33.9, 18.6.

Representative DCPD resin formulation

DCPD/ENB mixtures were first prepared at 5 wt% ENB by adding DCPD melted at 40-50 ^{o}C to a glass jar containing ENB and agitating until fully mixed. Photoresin was then formulated using the DCPD/ENB mixture as follows: to a 125 mL Thinky™ cup was added 20 mg of **HM** (0.030 mmol, 1 equiv), 200 mg of CQ (1.2 mmol, 40 equiv), 400 mg of EDAB (2.1 mmol, 70 equiv), and 140 mg of **NPPOC-TMG** (0.45 mmol, 15 equiv). CH_2Cl_2 was added in portions (~ 1 mL total volume) to fully homogenize these components, consistent with established literature

procedures.[1, 2] 20 g of DCPD/ENB mixture was then added, and the resin was agitated to homogenize. The photoresin was used immediately after preparation.

UV-Vis spectroscopic analysis of HM in the presence of Bronsted base.

Stock solutions of **HM**, norbornene monomer, and various Bronsted bases were prepared in 1,2-dichloroethane (DCE). For each experiment, these stock solutions were mixed immediately before measurements in 4 mL glass vials and diluted to 3 mL total volume with DCE. These mixtures were transferred to quartz cuvettes, and UV-Vis spectra were collected from 230-700 nm at 1 min intervals for a period of 30 min to monitor changes in electronic signatures associated with the **HM** catalyst.

Selective dual-wavelength olefin metathesis polymerization (SWOMP)

Cure and deactivation depth measurements. UV light from a high-powered LED (λ_{max} = 365 nm, 1400 mA, Thorlabs #M365LP1) was collimated using an aspheric condenser lens [Ø2″; f = 32 mm; numerical aperture (NA), 0.76; Thorlabs, ACL50832U] and focused with an adjustable collimation adapter (Thorlabs, SM2F). A commercial DLP projector (Optoma ML750) was retrofitted to emit blue light by disconnecting the green and red LEDs. Light from the blue projection system was passed through a biconvex (Ø2″; f = 100 mm; NA, 0.76; Thorlabs, LB1630) lens to reduce the focal distance and superimposed with the UV light using a longpass dichroic mirror (Ø2″, 425-nm cutoff; Thorlabs, DMLP425L). The intensity of the LEDs was controlled by adjusting the greyscale of an image in Microsoft PowerPoint. Light intensity at a given grey value was calibrated using a Thorlabs digital handheld optical power and energy meter console (PM100D). Spacers (two 0.635 mm stacked on each side) were clamped between two glass slides, and resin formulations were introduced between the slides via disposable pipette. The resin was

irradiated for 3 min at various intensities using the projector. After, the slides were separated, rinsed with CH_2Cl_2, and allowed to dry in an oven at 50 °C to minimize post-cure shrinkage. Final cure depth vales were measured using calipers. Similarly, deactivation depth was determined by using 0.635 mm spacers and subtracting the cure depth from 0.635 mm.

Greyscale and continuous SLA printing. Topologically complex objects were created by projecting greyscale images into resin formulations sandwiched between glass slides using spacers of 0.503 mm or 0.635 mm for 3 min. After removal of one glass slide, the resulting polymer film was rinsed with CH_2Cl_2 prior to post-curing in an oven initially for 2 h at 75 °C and then at 230 °C for an additional 30 min. Continuous SLA printing was performed using the setup described above, but glass slides were substituted with a vat possessing a transparent Teflon film (i.e., projection window). A build head (both projection window and build head were taken from a Kudo3D Micro SLA printer) attached to a programmable vertical stage (motorized linear stage, 100 mm travel, integrated controller, M4 and M6, catalog # FCL100) was used to continuously extrude the printed object from the vat. A Thorlabs CHROLIS 6-wavelength high-power LED source was used for continuous printing under high intensity irradiation.

Supplementary Characterization Data

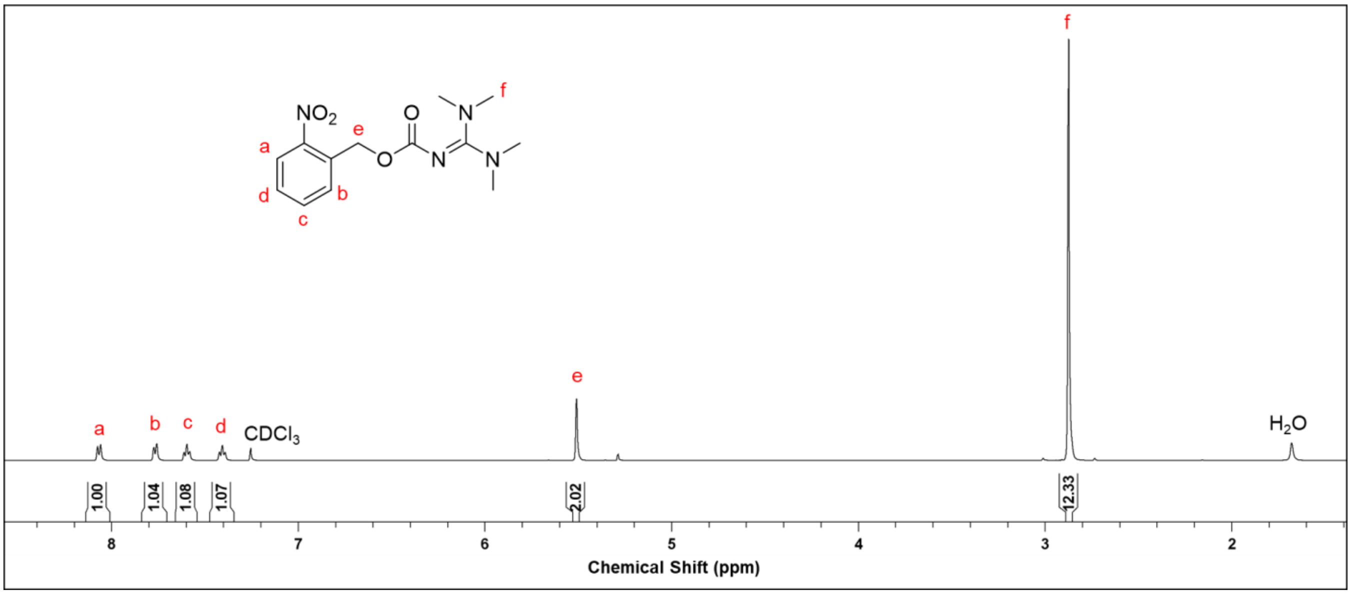

Figure S1. ^{1}H NMR spectrum of **NB-TMG** in $CDCl_3$.

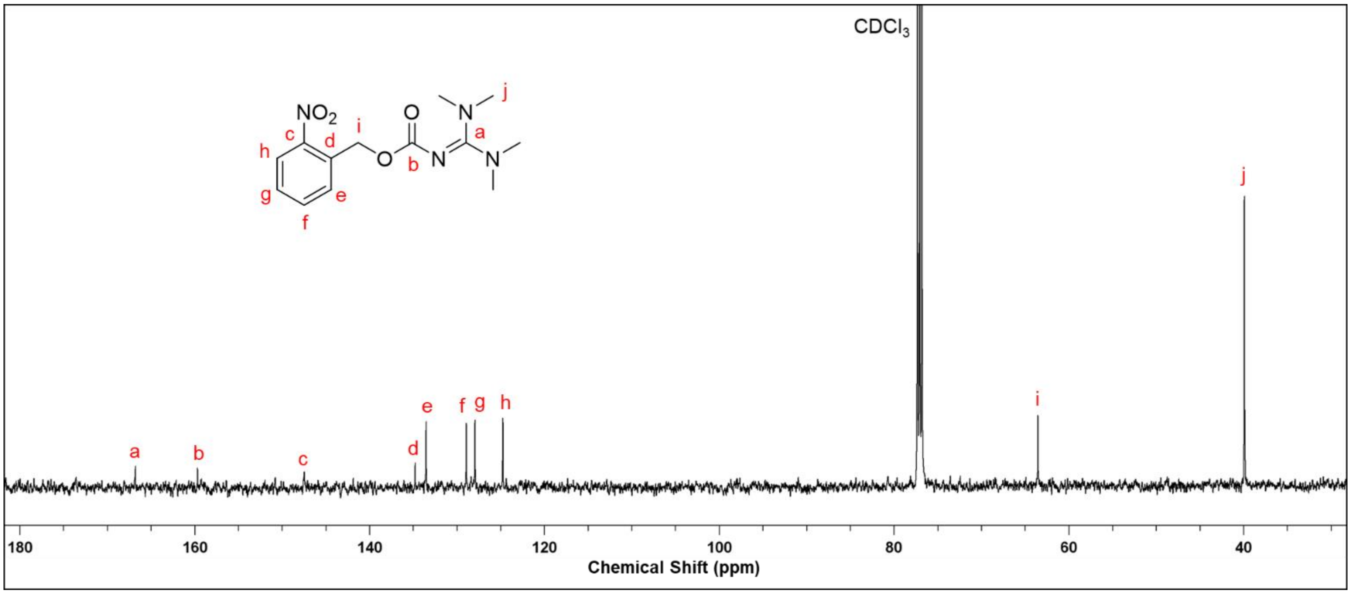

Figure S2. ^{13}C NMR spectrum of **NB-TMG** in $CDCl_3$.

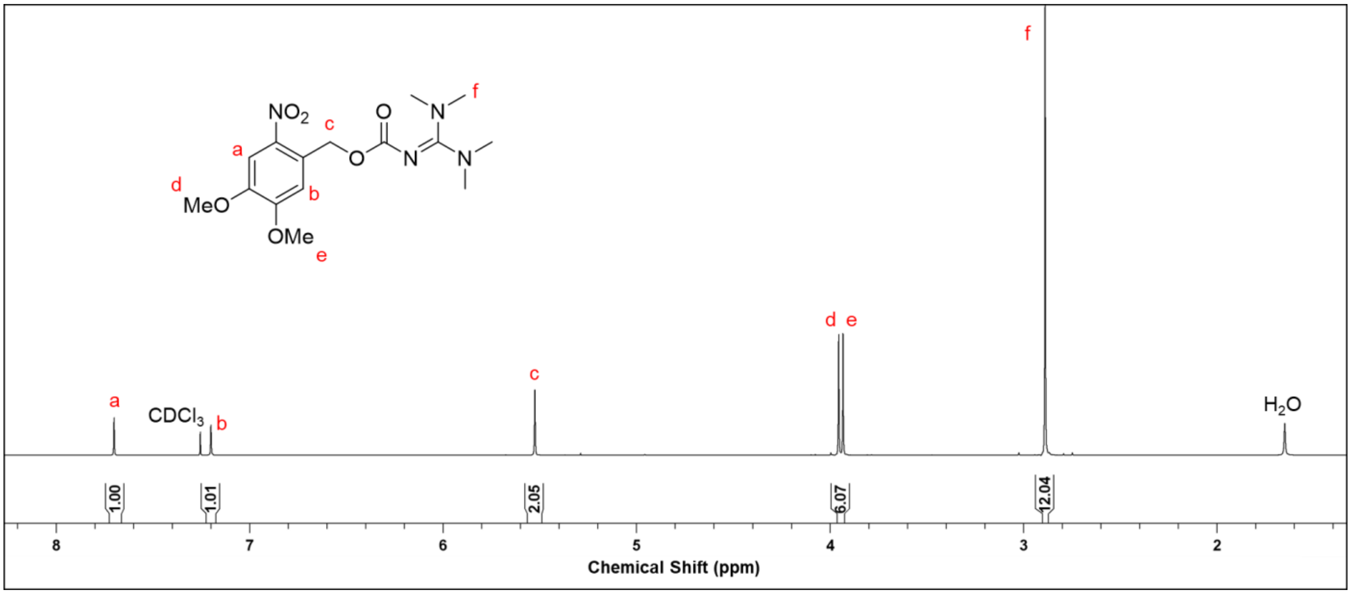

Figure S3. ^{1}H NMR spectrum of **NVOC-TMG** in $CDCl_3$.

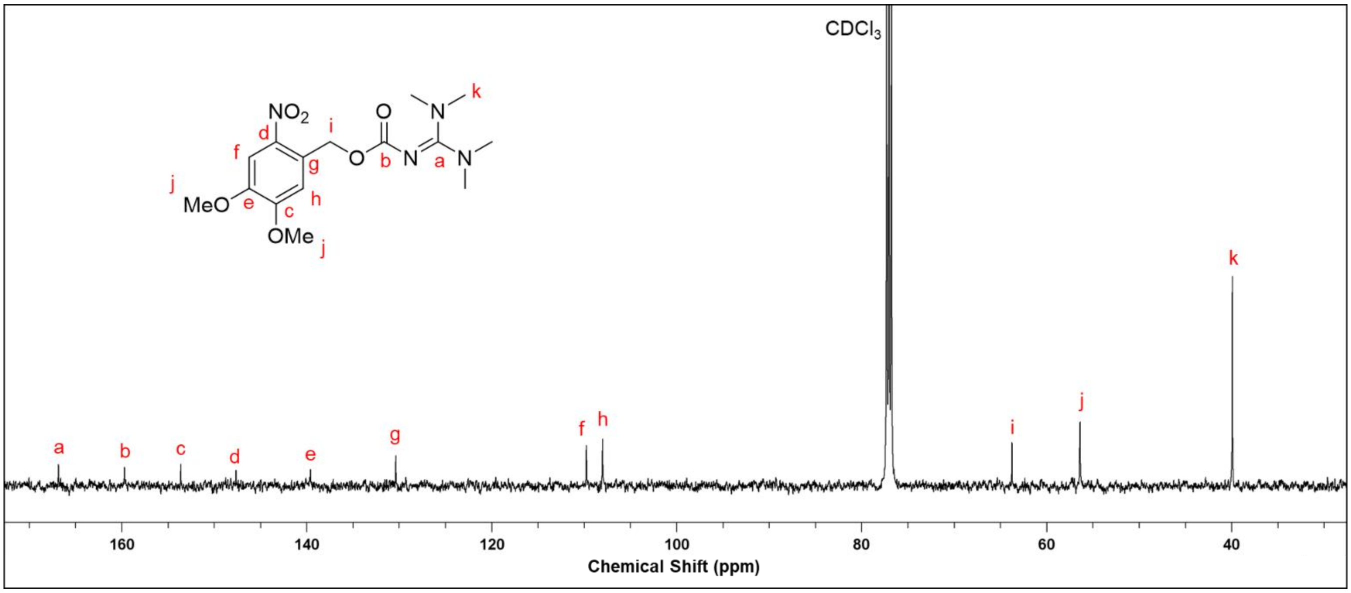

Figure S4. ^{13}C NMR spectrum of **NVOC-TMG** in $CDCl_3$.

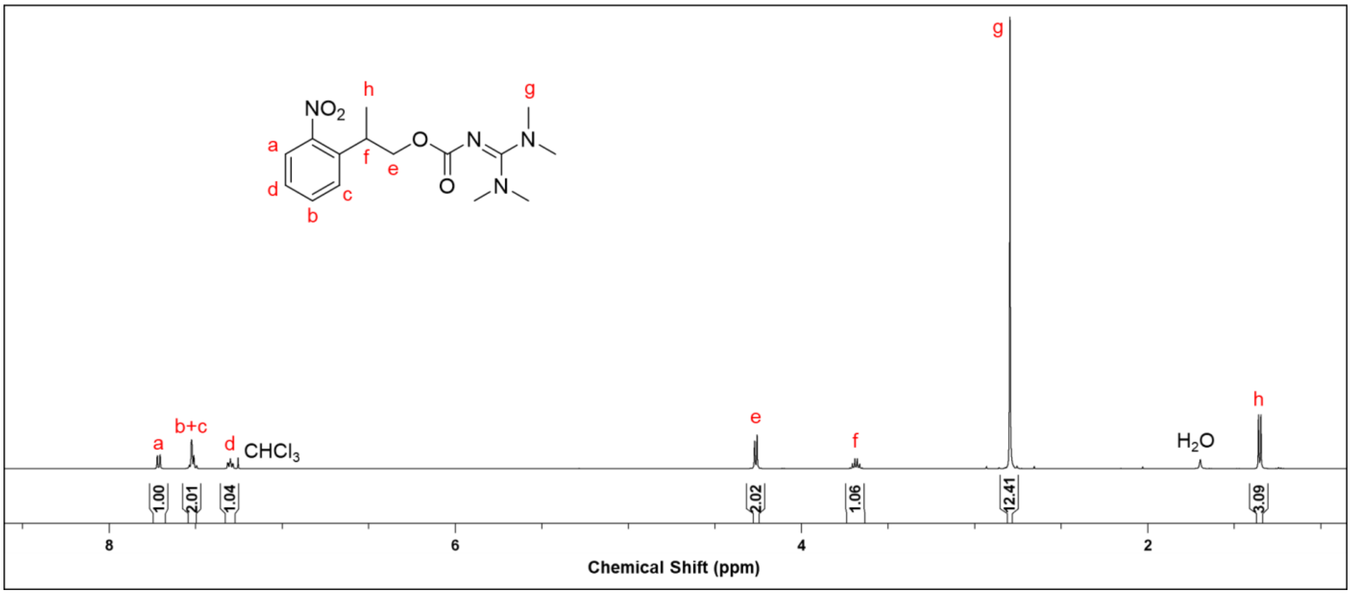

Figure S5. ^{1}H NMR spectrum of **NPPOC-TMG** in $CDCl_3$.

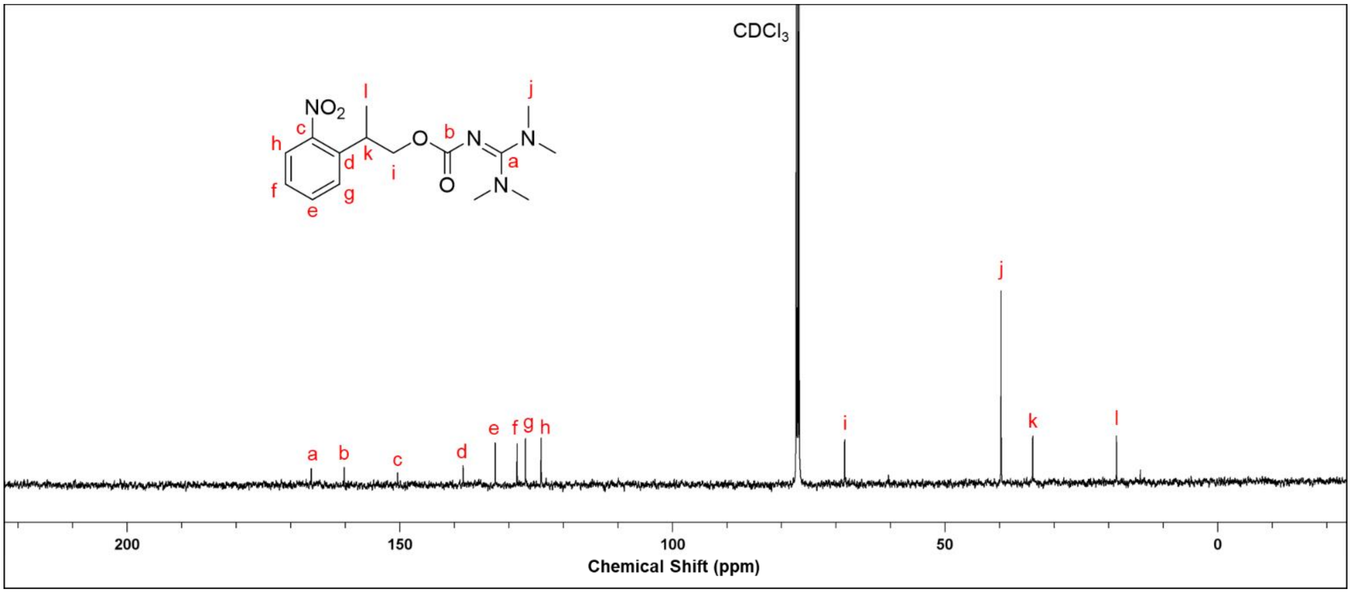

Figure S6. ^{13}C NMR spectrum of **NPPOC-TMG** in $CDCl_3$.

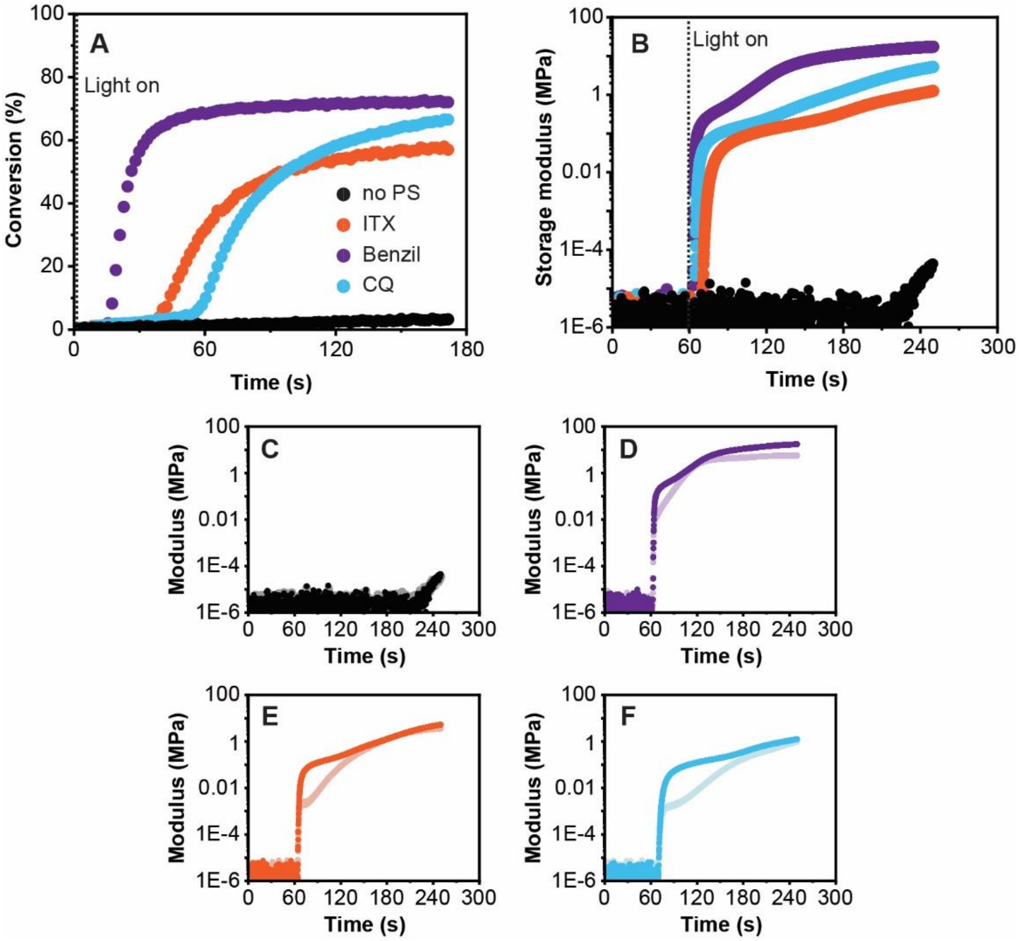

Figure S7. Evaluation of photosensitized ROMP of DCPD. **A)** Kinetics of polymerization as measured by FT-IR spectroscopy at 1573 cm^{-1} for **HM** alone (black circles), **HM**+Benzil+EDAB (purple circles), **HM**+ITX+EDAB (orange circles), and **HM**+CQ+EDAB (blue circles). **B)** Storage modulus vs time for the same polymerizations as measured by rheology. **C)** Storage modulus (black circles) and loss modulus (grey circles) vs time for the polymerization with **HM** alone. **D)** Storage modulus (purple circles) and loss modulus (light purple circles) vs time for the polymerization with **HM**+Benzil+EDAB. **E)** Storage modulus (orange circles) and loss modulus (light orange circles) for the polymerization with **HM**+ITX+EDAB. **F)** Storage modulus (blue circles) and loss modulus (light blue circles) for the polymerization with **HM**+CQ+EDAB. For the rheological experiments, the irradiation source (60 mW cm^{-1}) was turned on after 60 s for each wavelength. For FT-IR experiments, **HM** alone was irradiated at 365 nm (10 mW cm^{-1}), **HM**+Benzil+EDAB or **HM**+ITX+EDAB at 405 nm (10 mW cm^{-1}), and **HM**+CQ+EDAB at 475 nm (20 mW cm^{-1}). [DCPD]/[**HM**] = 5000 : 1 was used for these experiments with 0.2 wt % EDAB and 0.1% ITX or 1 wt% EDAB and 0.5 wt% benzil or 0.5 wt% CQ as appropriate.

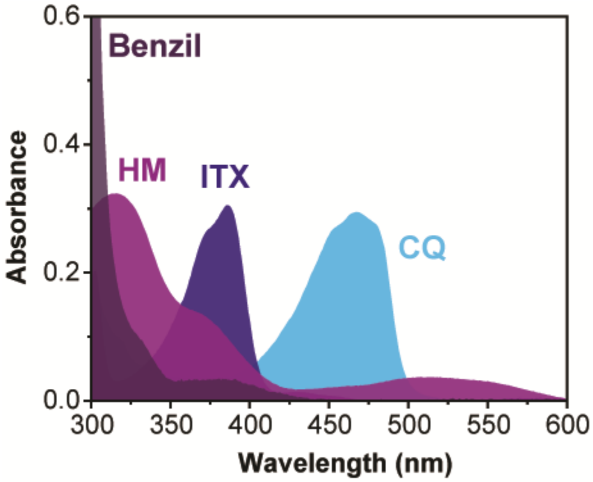

Figure S8. UV-Vis absorption spectra of **HM** and the various PSs used in this work.

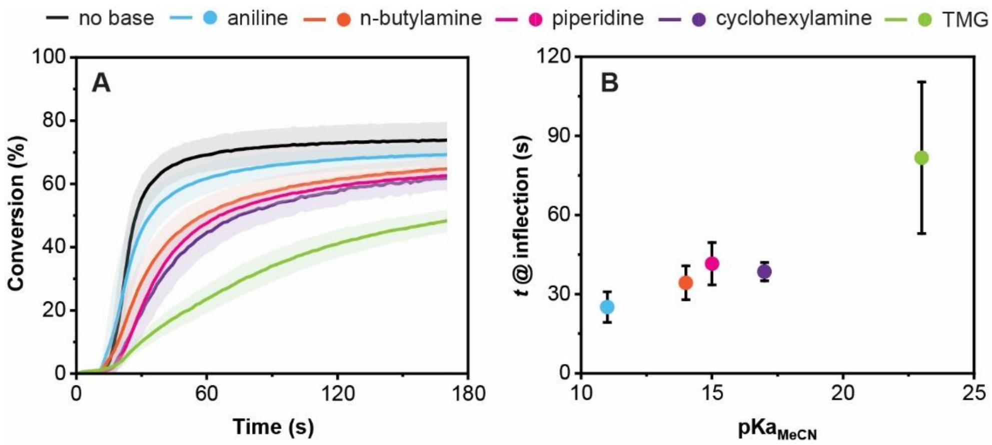

Figure S9. Evaluation of catalyst decomposition and polymerization deactivation by various nitrogenous bases. **A)** Averaged monomer conversion over time as measured by FT-IR spectroscopy at 1573 cm^{-1} in the polymerization of DCPD by **HM** in the presence of no added base (black trace) or 1 equiv of aniline (blue trace), n-butylamine (orange trace), piperidine (pink trace), cyclohexylamine (purple trace), or TMG (green trace). The shaded regions represent the standard deviation over n = 3 experiments. [DCPD]/[base]/[**HM**] = 5000 : 1 : 1 was used for these experiments with 1 wt% EDAB and 0.5 wt% benzil. **B)** Plot of the time at the inflection point of each trace in the monomer conversion plot as a function of the pKa of the base utilized. The colors of the individual points correspond to the colors of the traces in A. The error bars represent the standard deviation over n = 3 experiments.

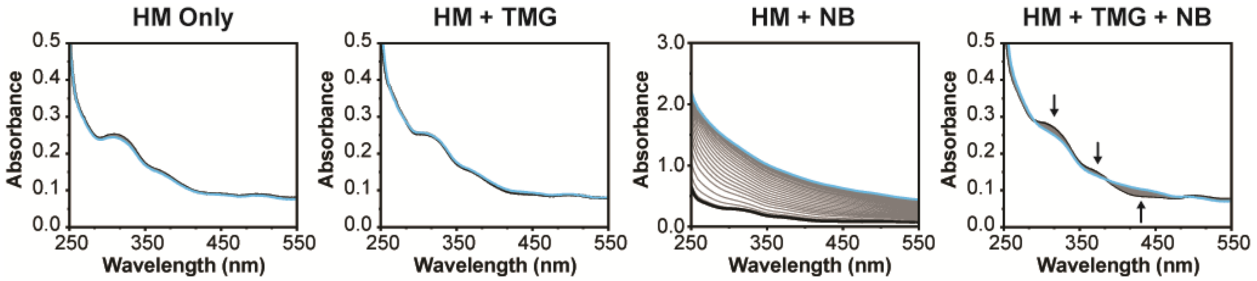

Figure S10. UV-Vis spectra of **HM** mixed with either NB, TMG, or NB and TMG in dichloroethane solution with irradiation at 365 nm. Spectra were collected at 1 min intervals for a total of 30 min. In each case, the black trace represents the initial timepoint and the blue trace the final. [**HM**] = 0.01 mg mL^{-1} was used for all experiments, with [NB]/[TMG]/[**HM**] = 5000 : 10 : 1. Note that a dramatic increase in absorbance was observed for the HM+NB sample under 365 irradiation due to P(NB) precipitation, signifying that polymerization was occurring.

Scheme S1. Proposed mechanism of catalyst deactivation by TMG.

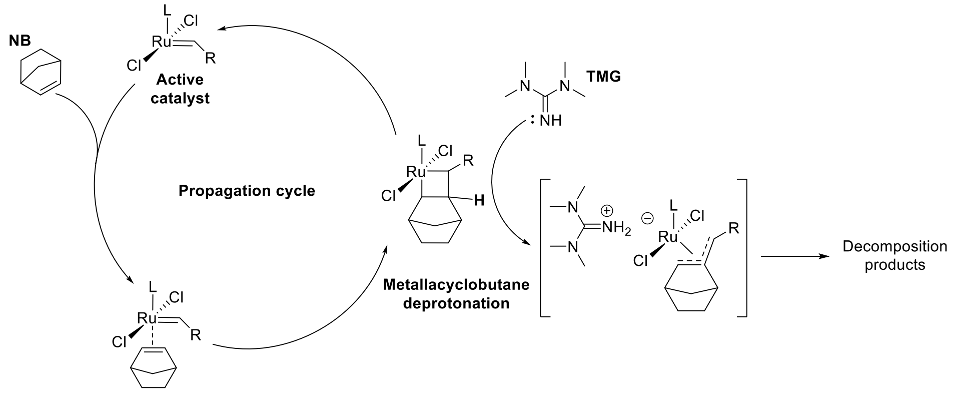

Scheme S2. Photolysis of the PBGs generates TMG along with other decomposition products.

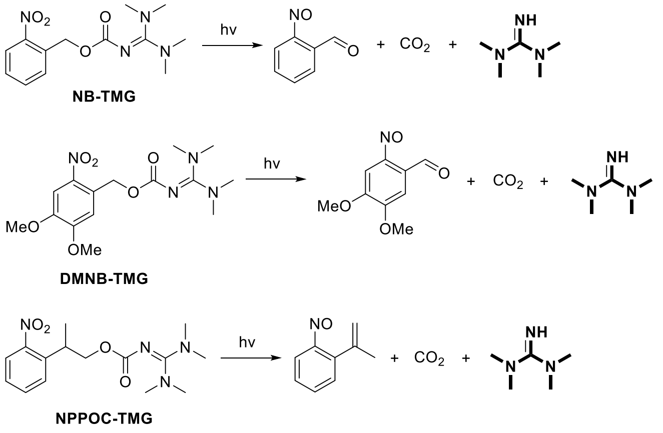

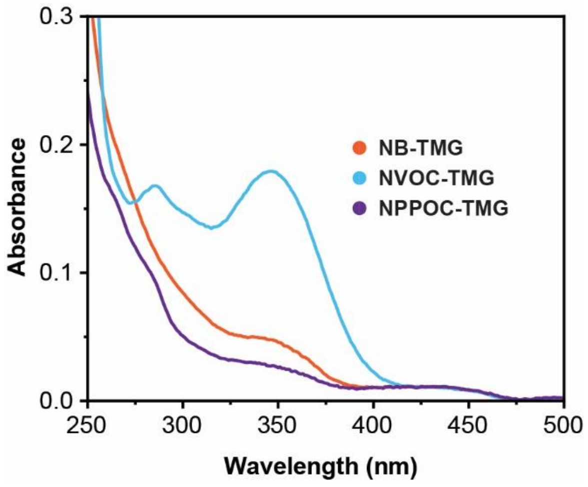

Figure S11. Absorbance spectra of **NB-TMG** (orange trace), **NVOC-TMG** (blue trace), and **NPPOC-TMG** (purple trace) at 0.01 mg mL^{-1} in dichloromethane solution.

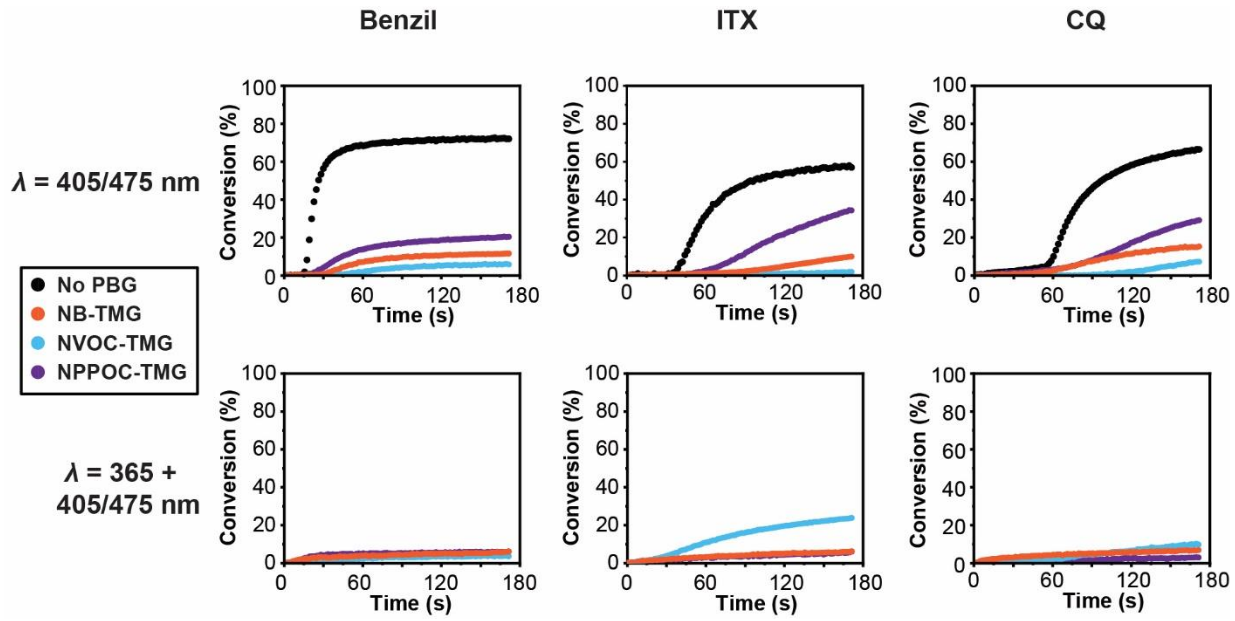

Figure S12. Kinetics of DCPD polymerization as measured by FT-IR spectroscopy at 1573 cm^{-1} for resins formulated using different PSs and PBGs. Samples were irradiated at either 405 nm for benzil and ITX or 475 for CQ (top row) to study initiation behavior or 365 nm + 405/475 nm to study polymerization deactivation (bottom row). The black curves correspond to control polymerizations carried out with the various PSs in the absence of PBG. For each plot, the appropriate PS was mixed with DCPD, **HM**, and either **NB-TMG** (blue circles), **NVOC-TMG** (orange circles), or **NPPOC-TMG** (purple circles). [DCPD]/[PBG]/[**HM**] = 5000 : 10 : 1 was used for these experiments, with 0.2 wt % EDAB and 0.1% ITX or 1 wt% EDAB and 0.5 wt% benzil or 0.5 wt% CQ as appropriate.

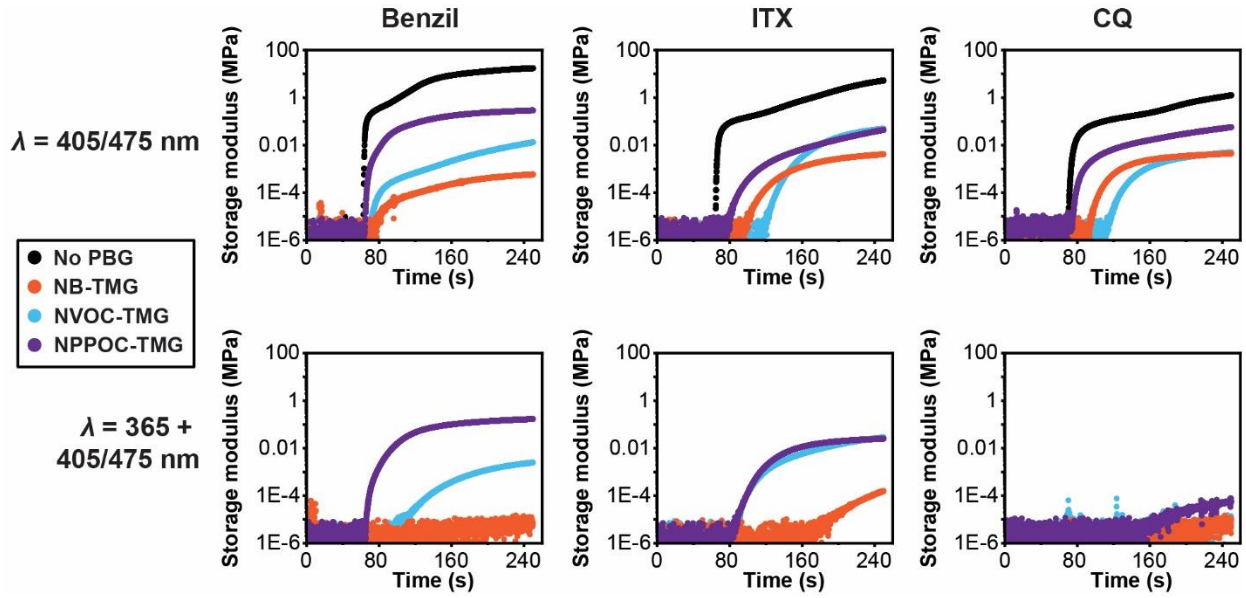

Figure S13. Storage modulus vs time for polymerizations using resins formulated with different PSs and PBGs. Samples were irradiated at either 405 nm for benzil and ITX or 475 for CQ (top row) to study initiation behavior or 365 nm + 405/475 nm to study polymerization deactivation (bottom row). The black curves correspond to control polymerizations carried out with the various PSs in the absence of PBG. For each plot, the appropriate PS was mixed with DCPD, **HM**, and either **NB-TMG** (blue circles), **NVOC-TMG** (orange circles), or **NPPOC-TMG** (purple circles). [DCPD]/[PBG]/[**HM**] = 5000 : 10 : 1 was used for these experiments, with 0.2 wt % EDAB and 0.1% ITX or 1 wt% EDAB and 0.5 wt% benzil or 0.5 wt% CQ as appropriate. The irradiation source (60 mW cm^{-1}) was turned on after 60 s for each wavelength.

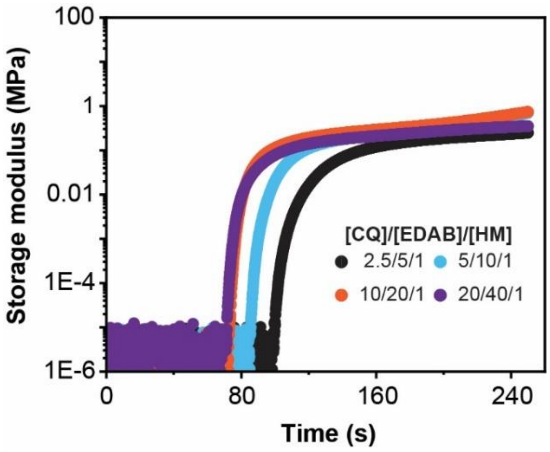

Figure S14. Storage modulus vs time for polymerizations using DCPD resins formulated with **HM** and different amounts of both CQ and EDAB. [DCPD]/[**HM**] = 5000 : 1 was used for these experiments, with [CQ]/[EDAB]/[HM] = 2.5 : 5 : 1 (black circles), 5 : 10 : 1 (blue circles), 10 : 20 : 1 (orange circles), and 20 : 40 : 1 (purple circles) by weight. The irradiation source (λ = 475 nm; 60 mW cm^{-1}) was turned on after 60 s.

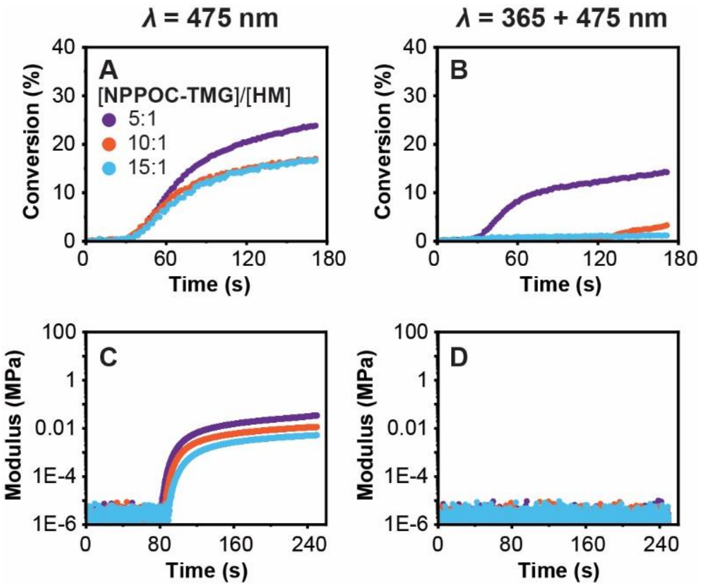

Figure S15. Kinetics of polymerization as measured by FT-IR spectroscopy at 1573 cm^{-1} for polymerization of DCPD by **HM** in the presence CQ, EDAB, and different amounts of **NPPOC-TMG** and irradiated at either **(A)** 475 nm or **(B)** both 365 nm and 475 nm. Storage modulus vs time for polymerizations using DCPD resins formulated with **HM**, CQ, EDAB, and different amounts of **NPPOC-TMG** and irradiated at either **(C)** 475 nm or **(D)** both 365 nm and 475 nm. For both experiments, 5 (purple circles), 10 (orange circles), or 15 (blue circles) equivalents of **NPPOC-TMG** were used relative to **HM**. [DCPD]/[**HM**] = 5000 : 1 was used for these experiments, with 2 wt% EDAB and 1 wt% CQ. For the rheological experiments, the irradiation source (60 mW cm^{-1}) was turned on after 60 s for each wavelength.

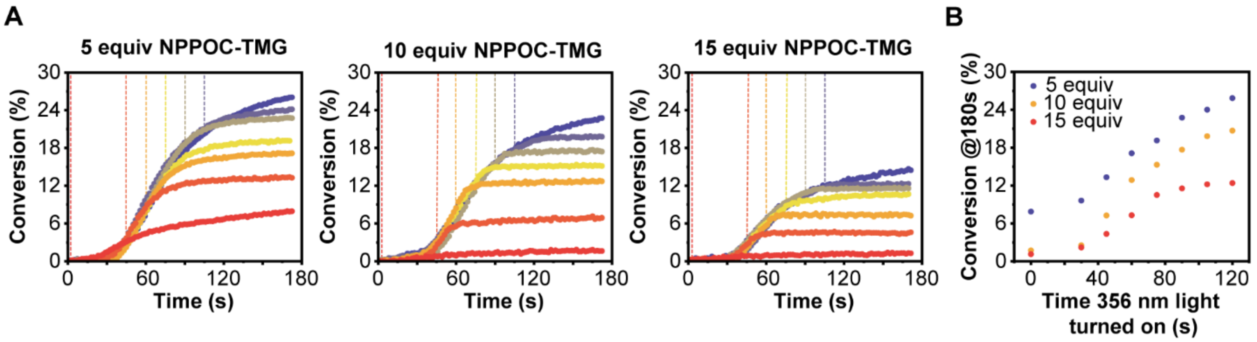

Figure S16. A) Kinetics of DCPD polymerization as measured by FT-IR spectroscopy at 1573 cm^{-1} for resins formulated using 5, 10, or 15 equiv of NPPOC-TMG relative to HM. For these experiments, 475 nm light was turned on at t = 0 to initiate polymerization and then 365 nm light was turned on at different time points (t = 0, 30, 45, 60, 75, 90, 105, or 120 s) to decompose the catalyst and thus deactivate polymerization. Monomer conversion significantly slowed or stopped altogether shortly after the 365 nm light was turned on. B) Plateau (ultimate) conversion as a function of the time that the 365 nm light was turned on during the experiments.

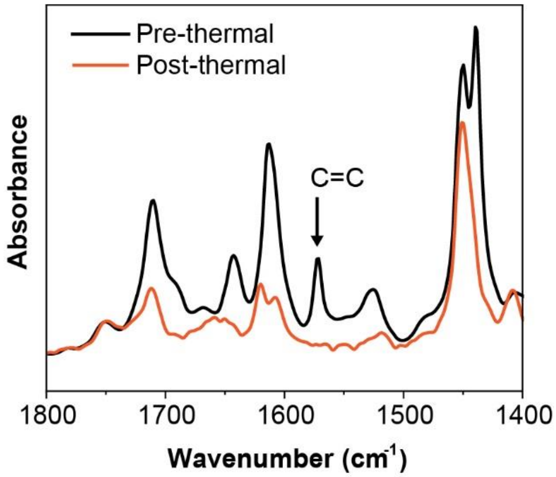

Figure S17. FT-IR spectra of a part produced via SWOMP immediately after photopolymerization (black trace) and following a thermal post cure at 250 °C for 30 min (orange trace). The signal at 1573 cm^{-1} corresponds to residual unreacted alkenes derived from residual DCPD monomer that become fully consumed during the post-curing process.

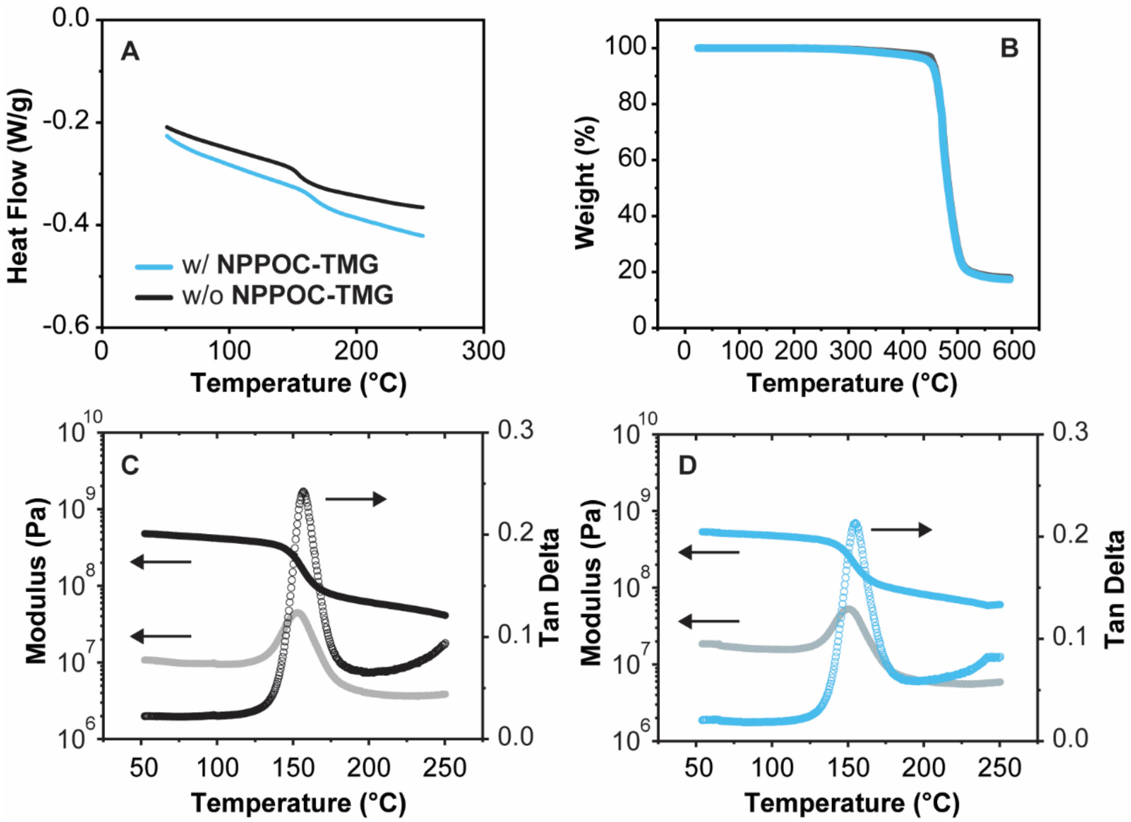

Figure S18. Thermal analysis of PDCPD films prepared by photo-activated ROMP using the optimized resin formulation under 475 nm irradiation. **A)** DSC of PDCPD films prepared using resins with (blue trace) and without (black trace) NPPOC-TMG. The calculated T_g values were 164 °C and 153 °C, respectively. Shown is the 2nd heating cycle, wherein the temperature was increased from 50-250 °C at 10 °C min^{-1} under N_2. **B)** TGA of the same films prepared using resins with (blue trace) and without (black trace) NPPOC-TMG. Note that the traces are overlapped. A heating rate of 20 °C min^{-1} was used. Complete DMA data for the same films prepared using resins with **(C)** and without **(D)** NPPOC-TMG. Shown are storage modulus (closed circles, darker traces), loss modulus (closed circles, lighter traces) and tan delta (open circles). [DCPD]/[NPPOC-TMG]/[**HM**] = 5000 : 15 : 1 was used for these experiments, with 2 wt% EDAB and 1 wt% CQ.

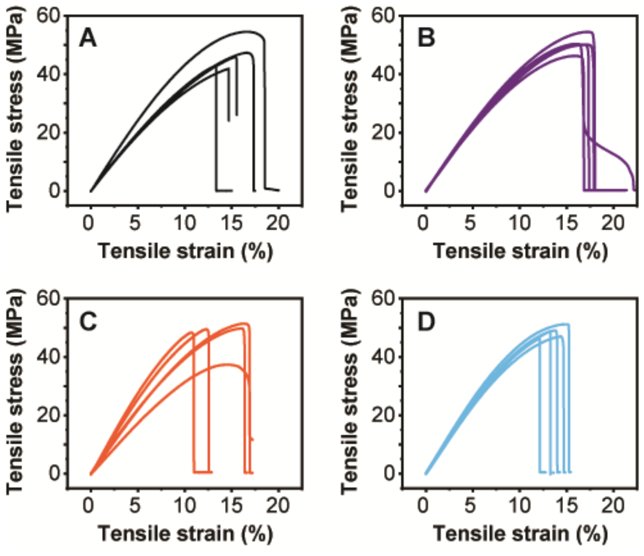

Figure S19. Stress vs strain curves for PDCPD dogbones prepared by photo-activated ROMP using the optimized resin formulation but with either **(A)** 0 equiv, **(B)** 5 equiv, **(C)** 10 equiv, or **(D)** 15 equiv of **NPPOC-TMG** relative to **HM**. [DCPD]/[**HM**] = 5000 : 1 was used for these experiments, with 2 wt% EDAB and 1 wt% CQ.

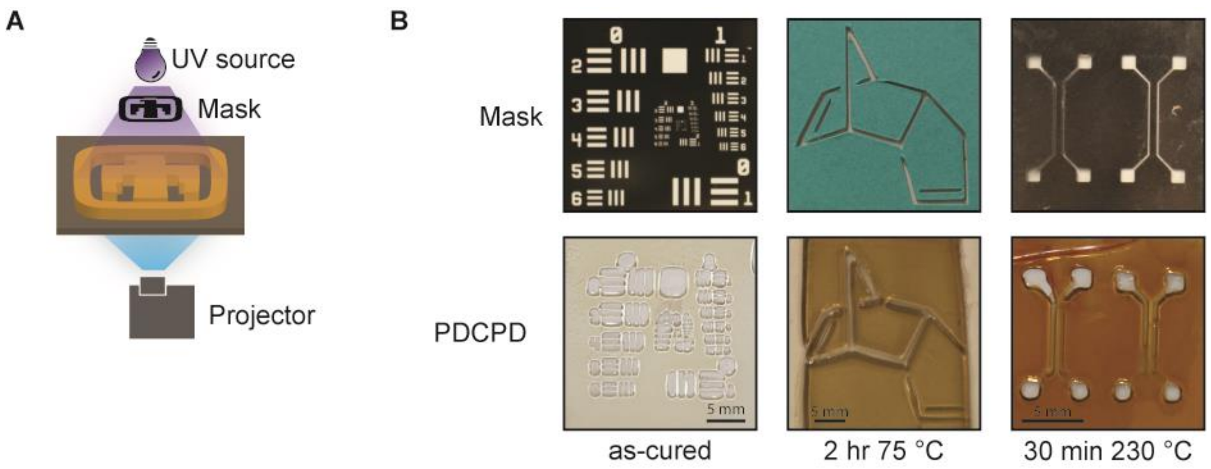

Figure S20. Stereolithography using dual-wavelength approach. **A)** Schematic of stereolithographic setup, wherein the resin is illuminated with a constant background of blue light from below and patterned UV light from above. Resin curing is inhibited in the regions where the UV light is present. **B)** Optical photographs of photomasks and corresponding cured resins obtained by this process. Images from different stages of the printing and post cure process were included to highlight the changes in appearance of the polymer films.

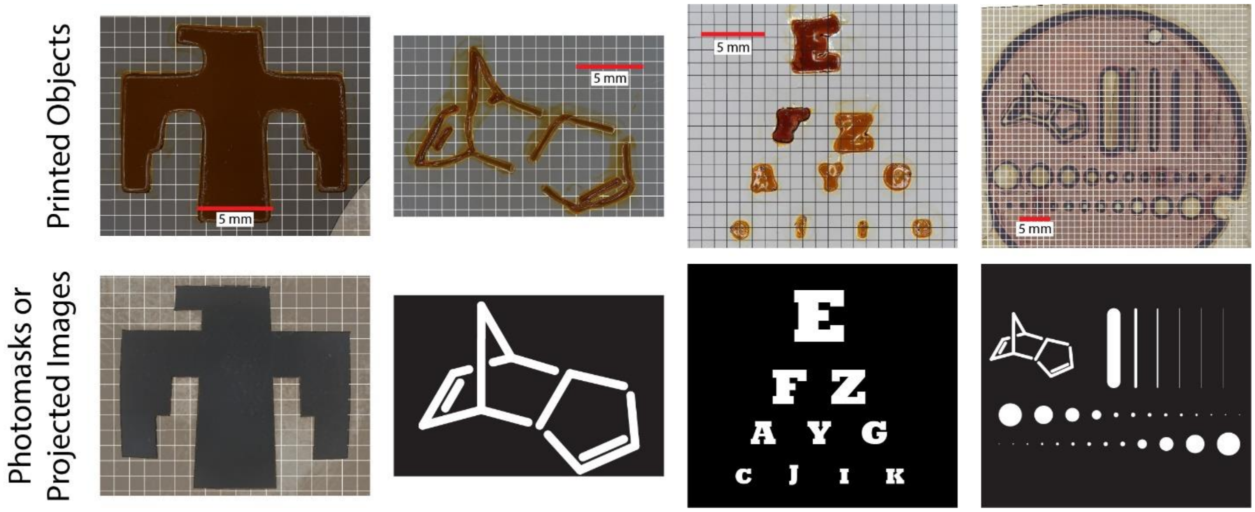

Figure S21. Additional 2D objects produced using a photomask and 475 nm irradiation from a high-intensity light source (two leftmost images), an image projected with a blue light emitting projector (second to last image), and 475 nm irradiation from a high-intensity light source and a 365 nm light engine to pattern UV light similar to the setup shown in Figure S20 (rightmost image). These objects showcase sub-mm x,y resolution can be achieved using either a single-wavelength or a dual-wavelength approach. Grids represent 1 mm × 1 mm squares. [DCPD]/[**NPPOC-TMG**]/[**HM**] = 5000 : 15 : 1 was used for these experiments with 1 wt% CQ and 2 wt% EDAB.

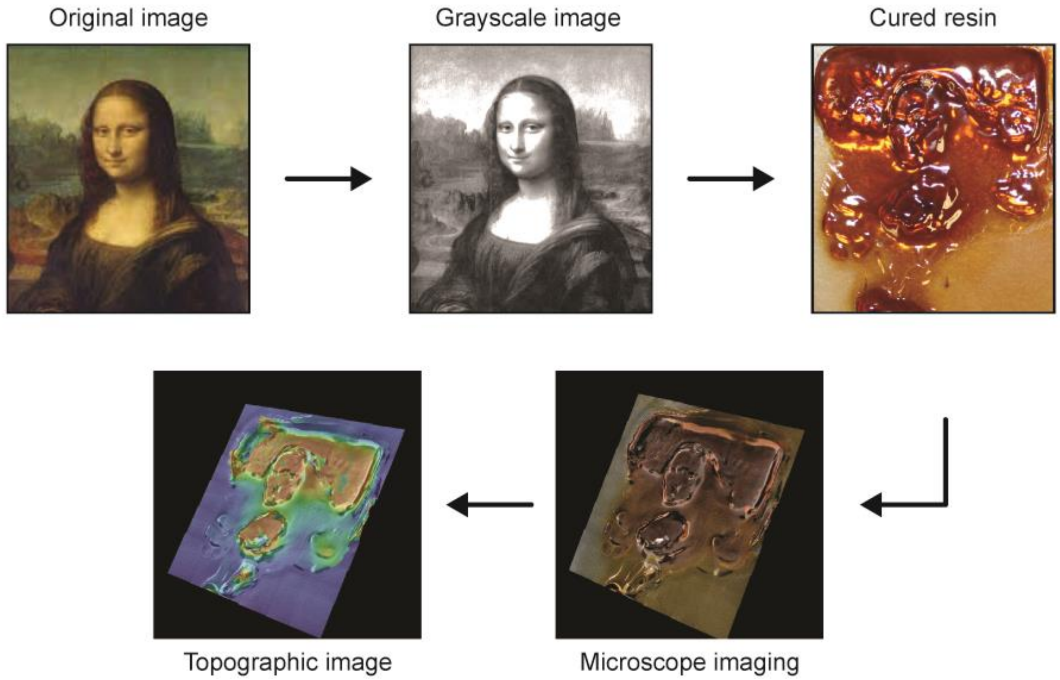

Figure S22. Stereolithography using a dual-wavelength approach enables facile translation of digital images to cured parts. White regions on the grayscale images yield the shallowest deactivation depths, while black one result in the largest deactivation depths (and thus no cure). This experiment highlights the ability to produce complex topological objects from a single exposure.

[1] O. Eivgi, A. Vaisman, N. B. Nechmad, M. Baranov, N. G. Lemcoff, ACS Catalysis 2020, 10, 2033.
[2] O. Eivgi, A. Vaisman, N. G. Lemcoff, ACS Catalysis 2021, 11, 703.
